# Community-Based Development of Tools to Assess Firearm-Related Attitudes and Beliefs: Associations with Firearm-Related Practices, Social Influences, and Violence Exposure

**DOI:** 10.1007/s10900-026-01587-6

**Published:** 2026-06-05

**Authors:** Krista R. Mehari, Albert D. Farrell, Jasmine N. Coleman, Lindiwe S. Mayinja, Colleen S. Walsh, Phillip N. Smith

**Affiliations:** 1 College of Human Medicine, Charles Stewart Mott Department of Public Health, Michigan State University, MI Flint, USA; 2 Department of Psychology, Virginia Commonwealth University, Richmond, VA USA; 3 Department of Psychology, University of Tennessee-Knoxville, Knoxville, TN USA; 4 Peabody College of Education and Human Development, Department of Psychology and Human Development, Vanderbilt University, Nashville, TN USA; 5 The Henry M. Jackson Foundation for the Advancement of Military Medicine, Inc., Maryland Bethesda, United States; 6 Department of Psychology, University of South Alabama, Mobile, AL USA

**Keywords:** Guns, Policy, Norms, Violence, Social influences

## Abstract

The purpose of this study was to evaluate four novel measures of firearm-related attitudes and beliefs developed through participatory action research, and to determine their associations with firearm-related practices and violence exposure. Participants were a pilot sample of 550 U.S. adults (51% female; 65% White; 16% Latine; 13% Black; *M*_age_ = 48.3) and a nationally representative cross-validation sample of 1,674 U.S. adults (52% female; 61% White; 18% Latine; 12% Black; *M*_age_ = 47.4). Competing models of the structure of each measure were evaluated based on analyses of data from the pilot sample. This identified a single factor for General Attitudes Toward Firearms measure, three factors for a Beliefs About Defensive Firearm Use measure (Respect Enforcement; Defensive Escalation; and Compensatory Force); two factors for a Prescriptive Norms for Threat Response measure (Defense and Protection; Reputation and Respect); and two factors for an Attitudes Toward Firearm-Related Policy measure (Negative Attitudes Toward General Firearm Policies and Positive Attitudes Toward Specific Firearm Policies). Analyses of the cross-validation sample replicated this structure and provided support for strong measurement invariance across sex and ethnicity. The construct validity and utility of these measures was supported by their patterns of relations to firearm-related practices (ownership, carrying, and storage); involvement in firearm-related violence; and family and friend firearm-related influences (e.g., friends’ firearm carrying; household presence of a firearm; close family members’ and friends’ injury or death due to violence or suicide). These new measures provide promising tools to advance firearm-related injury prevention research.

**Supplementary Information:**

The online version contains supplementary material available at https://doi.org/10.1007/s10900-026-01587-6.

There is a strong need for measures that assess individual factors that predict firearm-related practices, behaviors, and violence exposure. Such measures could be particularly useful for identifying key areas on which to focus firearm-related injury prevention efforts. Potential factors include attitudes—positive or negative evaluative judgments [1]—and beliefs—cognitive structures that assign objects to probability categories [2]. Despite their potential value, there are currently no well-established measures for assessing firearm-related attitudes and beliefs. The purpose of this study was to rigorously evaluate the structure and construct validity of four measures developed to assess key constructs identified through participatory action research conducted with firearm owners and individuals living in communities with high rates of firearm violence. These included Prescriptive Norms for Threat Response; Beliefs About Defensive Firearm Use; General Attitudes Toward Firearms; and Attitudes Toward Firearm-Related Policy.

The focus of current firearm-related attitudes and beliefs measures has been determined primarily by researchers, rather than through partnerships with individuals who own and use firearms, or who are members of communities heavily impacted by firearm-related violence [3, 4]. This is a serious limitation because of the deep cultural divide between public health researchers, who tend to be more politically liberal and less religious than the general public, and individuals who own and use firearms, who tend to be more theologically and politically conservative and more religious than the general public [5]. Thus, firearm-related measures created solely by researchers may assess an overly narrow range of attitudes and beliefs and include item wording that reflects a particular bias. Moreover, researchers have often created measures for specific studies without providing much information about how constructs were identified and defined, how item pools were developed or refined, or the construct validity of the measure [6–8].

According to social-cognitive theory, attitudes and beliefs are influenced by individuals’ experiences through a process of interpretation and evaluation, which creates internalized standards for behavior. These internalized standards, including valence judgments and injunctive norms, in turn, predict individuals’ behaviors—from personal ownership and firearm-related practices to voting patterns—that shape their experiences [9]. A clear implication is that firearm-related attitudinal and beliefs measures should be associated with both experiences and behaviors.

Currently, the most frequently used measures of firearm-related attitudes focus on attitudes about firearm-related policies and laws [10–12]. Research in this area has primarily focused on identity-related factors such as sex and race or ethnicity, with men and White adults generally being more opposed to restrictive firearm-related laws [11]. Individuals with personal or household firearm ownership are less likely to support restrictive firearm laws in general and more likely to oppose specific laws, such as semi-automatic weapons bans or universal background checks for firearm purchases [10, 12–14]. Despite the link between identity and firearm ownership, few studies have explored the relation between firearm policy attitudes and firearm-related behaviors or violence exposure. One study using a nationally representative sample of U.S. adults found that prior firearm-related victimization did not predict attitudes toward firearm-related legislation [12].

Other commonly used measures assess general beliefs about firearms, including ownership rights, the value of firearms for protection, and the impact of firearms on crime [3, 15, 16]. Research in this area has indicated that firearm owners are more likely to believe that firearms make them safer and are an effective means of defense [8, 15], whereas non-owners are more likely to believe there is risk or danger associated with firearm ownership [7]. One study found that firearm owners with stronger beliefs about firearm-related risks were more likely to engage in secure storage practices [17].

Another set of beliefs linked to firearm-related violence, but not explicitly connected to attitudes about firearms, is normative beliefs about the appropriate level of violence in response to perceived threat or provocation. Such beliefs are often related to a need to protect oneself or close others, to preserve one’s image or reputation, and to retaliate against physical threats or disrespect. This construct is sometimes called “culture of honor” or masculine honor beliefs when describing White men in the South [18] and “code of the street” when describing Black boys and men in urban areas [19]. A few studies have found that masculine honor norms are associated with firearm ownership [20, 21]. Stronger beliefs reflecting the need for retribution or retaliation and for courage in a fight have been found to predict violent behavior, regardless of race or gender [22, 23]. Research on “code of the street” and “culture of honor” has generally been conducted in siloes based on race and urbanicity, despite evidence that they are likely the same phenomenon, with similar etiologies and patterns of relations to homicide and violent injury [24].

A very small body of work has examined beliefs about defensive firearm use, generally relying on single-item measures in large epidemiological datasets. In one such study, individuals with firearms in their home were more likely to endorse beliefs supporting the use of firearms against others, such as an intruder [25]. Another study found that firearm owners were more likely to endorse beliefs supporting shooting an intruder as well as the perceived right to kill [8].

## Purpose of the Current Study

The purpose of this study was to develop measures assessing a range of attitudes and beliefs about firearms in partnership with communities who use firearms and are impacted by firearm violence, and to determine how these measures are related to firearm-related practices, influences, and violence involvement. We seek to address several key limitations that exist in the field. First, the lack of rigorously developed and evaluated measures of attitudes about firearms is a major gap in the field. In addition, a key limitation of existing measures has been their development without explicitly incorporating the experiences and perspectives of communities that own and use firearms—this approach has likely resulted in an overly narrow focus on specific constructs without exploring potential multidimensional components of firearm-related beliefs. Furthermore, there is little information about the validity of existing attitudinal measures in terms of their relation to prior firearm-related exposures and current firearm-related practices. The present study used participatory action research with firearm owners and individuals living in high-violence communities to develop four measures: Prescriptive Norms for Threat Response, Beliefs About Defensive Firearm Use; General Attitudes Toward Firearms; and Attitudes Toward Firearm-Related Policy. Initial pools of items were developed and analyses were conducted to establish their factor structure and to examine measurement invariance across ethnicity and sex. Their construct validity was then evaluated by examining their relations with demographic factors, firearm-related practices, and exposure to firearm-related violence and injury.

Our primary hypotheses were: (1) the factor structures would be invariant across ethnicity and sex; and (2) the measures would have discriminant validity as indicated by small to moderate correlations with each other. We also hypothesized that (3) demographic factors would predict firearm-related attitudes and beliefs, with general trends such that men and White adults would have more positive attitudes toward firearms and their use and less supportive attitudes toward restrictive firearm-related policies. We hypothesized that (4) attitudes and beliefs would be associated with firearm-related practices, influences, and violence exposure. The full list of all preregistered hypotheses is available in Supplemental materials (Appendix A) and on the Open Science Framework. Analyses for which we were not able to develop hypotheses based on theory or empirical work were considered exploratory.

## Method

### Participants

A pilot sample of U.S. adults was recruited to provide a derivation sample for initial analyses of the measures. The analysis sample included 550 participants after excluding 2 who answered 11 items or less, and 11 who answered more than 20 questions per minute (over two standard deviations above average). Ages were from 18 to 87 years (M = 48.3, SD = 17.3). The sample included 283 women, 259 men, and 8 participants who did not identify their sex; 64.5% self-identified as White, 15.6% as Hispanic/Latino/a, 13.1% as Black, 2.9% as Asian, 2.7% as multiracial, and 1.1% as another race. About one-third (34.9%) were college graduates; another 42.9% had some vocational, technical school, or college education. Household incomes were less than $30,000 for 21.1% of the sample, $30,000 to under $60,000 for 29.5%, and $100,000 or more for 25.8%. The majority (85.5%) were from metropolitan areas; 32.9% from the South, 29.5% from the Midwest, 25.1% from the West, and 12.5% from the Northeast.

An independent, nationally representative sample of adults was recruited to provide a basis to cross-validate the analyses of the pilot sample and to evaluate the construct validity of the measures. An initial sample of 1,899 was reduced to 1,674 participants after excluding 87 participants who did not provide consent, 44 who provided duplicate responses, 63 who opened but did not complete the survey, 2 who answered 23 items or less, and 29 who completed the survey too quickly (over two standard deviations above average). The final sample was 51.8% female; 61.4% self-identified as White, 18.1% as Hispanic/Latino/a, 12.1% as Black, 4.1% as Asian, 3.3% as multiracial, and 1.1% as another race. Ages ranged from 18 to 93 years (M = 47.4, SD = 17.5). Over one-third (38.5%) were college graduates; another 37.4% had some vocational, technical school, or college education. Household incomes were less than $30,000 for 21.9% of the sample, $30,000 to under $60,000 for 25.4%, and $100,000 or more for 27.9%. The majority (86.7%) were from metropolitan areas; 33.6% were from the South, 25.7% from the Midwest, 28.3% from the West, and 12.4% from the Northeast. Sampling weights adjusted for sample selection probabilities across 48 strata (including the intersections of race/ethnicity, age, education, and sex).

### Procedures

All participants were recruited through the AmeriSpeak Panel, a probability-based U.S. sample of over 41,000 participants operated by NORC at the University of Chicago. The NORC National Frame sampling approach resulted in a panel within 1–2% points of U.S. Census data (by race/ethnicity, age, region, and rurality/urbanicity) [26]. Participants could complete the survey in English or Spanish, over the internet or telephone, and received AmeriSpeak (cash-equivalent) points. The median survey completion time was 22 min.

### Development of Firearm-Related Beliefs and Attitudes Measures

Items representing key domains of firearm-related attitudes and beliefs were developed through a participatory action approach [5]. Because searches of the relevant literature did not reveal any existing measures of these constructs, our team developed measures based on the following steps. First, descriptions of the constructs were refined through discussions among three advisory boards that included community leaders working with youth and young adults in high-violence areas; young adults living in high-violence communities; individuals identifying as firearm owners; and community research assistants. These discussions were informed by themes that emerged in over 200 qualitative interviews with firearm owners and individuals living in areas of high community violence. For each construct, potential subdomains were identified, including components of the construct that seemed to be important to assess.

Item pools for measures of firearms related attitudes and beliefs were developed collaboratively by a team of investigators and community research assistants based on themes from the qualitative interviews and the advisory board meetings, and were supplemented by literature reviews conducted by graduate students. Item pools were refined through an iterative process in which team members ranked items by importance and suggested alternate wording. The most important items by subdomain were then selected and revised. These items were then brought to the advisory board members, who engaged in cognitive testing of the items. Specifically, they reported their reactions to the items, what they thought the items were trying to assess, perceived bias in wording, and how they responded and why. Extensive wording revisions were made in response to this feedback, with some exceptions in the cases of competing feedback. For example, whereas community leaders indicated that the General Attitudes Toward Firearms measure had too many positive items, the firearm owners indicated that it had too many negative items. However, when asked to identify the positive and negative items, members agreed there was an equal amount of positive and negative items, so we did not change the balance of items. We conducted a Flesch-Kincaid readability analysis of the full pool of items for all four measures to estimate the readability of the items [27, 28]. The items fell within the sixth-grade level in the U.S.-based education system (i.e., 11–12 years old) and had a reading ease score of 69, consistent with appropriate use for the general public.

The team developed an initial pool of 12 items for a General Attitudes Toward Firearms measure; 22 items for a Beliefs About Defensive Firearm Use measure to assess beliefs justifying firearm use in general and in specific situations; and 9 items for a Prescriptive Norms for Threat Response measure to assess perceptions of cultural norms about protecting oneself, family, and property, as well as defending one’s reputation (see Tables C-2, D-2, and E-2 in Supplemental materials for the initial pool of items). Participants were given the prompt “How much do you agree with the following statements?” for the first two measures, and “People in different places believe different things. How much do the people around you agree with the following statements?” for the prescriptive norms measure. Participants rated each item on a 5-point scale: 1 = *Strongly disagree* to 5 = *Strongly agree*.

The initial pool of items for an Attitudes Toward Firearm-Related Policy measure included 11 items to assess attitudes toward current and potential firearm-related laws and policies, and 9 items to assess approval or disapproval of specific firearm-related laws and policies that have been proposed or put into place (see Table F-2 in Supplemental materials). Participants rated these items on the same 5-point scale as the General Attitudes Toward Firearms items and rated the degree to which they supported or opposed the 9 policy items on a 5-point scale: 1 = *Strongly oppose* to 5 = *Strongly support*.

### Measures

#### Firearm-Related Violence, Injury, and Exposure

Lifetime firearm-related perpetration was coded as present based on responding *Yes* to either of two questions: “Have you ever used a firearm to defend yourself or someone else against a person or group of people?” and “Other than to defend yourself, have you ever used a firearm against someone?” Lifetime firearm-related victimization was coded as present based on responding *Yes* to one or more items asking about any lifetime occurrence of having “been shot at with a firearm,” “been threatened with a firearm,” or “had an accident or an injury from a firearm where any part of your body was hurt?” in which the injury was intentional and caused by someone else. Lifetime witnessing of firearm-related violence was coded as present based on affirmative responses to the question, “In your lifetime, have you ever seen someone else get shot at with a firearm?”

#### Firearm-Related Practices

Responses to the item “Do you currently have a firearm or firearms?,” which included three choices (*No*,* Yes*, and *There’s a firearm where I live*,* but it’s not mine*) were coded as *No Access*,* Personal Ownership*, and *Household Ownership*, respectively. Carrying a firearm was coded Ever/Never based on responses to the question “Do you ever carry a firearm?” Coding of unsecured firearm storage practices was based on how and where participants stored their firearm(s). It was coded *Yes* (unsecured) for participants who reported that they both kept a firearm loaded *and* in an easily accessible location (e.g., “sitting on a table or shelf by the door”). Storage was coded *No* (secured) for participants who responded that they kept their firearm unloaded, with ammunition stored separately, or with device locks (trigger locks, barrel, or action locks); or who did not report storing their firearm in an easily accessible location.

#### Firearm-Related Friend and Family Influences

Growing up with firearms in the home was based on the question, “Were there firearms in the home where you grew up?;” *No* responses were coded as *No*, and *Sometimes* or *Yes* responses were coded as *Yes*. Friends’ firearm carrying was coded *Yes* if they reported at least some of them did so. Eight items assessed family and friends’ history of violence and suicide. Family and friends’ history of violence, respectively, were coded as *Yes* if the participant indicated that a family member or close friend had been injured or killed due to violence. Family and friends’ history of suicide, respectively, were coded as *Yes* if the participant indicated that a family member or close friend had tried to kill themselves or had died by suicide.

#### Demographic Measures

Race or ethnicity was provided by AmeriSpeak based on existing panel data in the following categories: White, Black, Other, Hispanic, multiple races, and Asian. Sex at birth was assessed by a single-item indicator, “When you were born, what did they say your sex was?” with response options of male, female, or intersex.

### Transparency and Openness

We report how we determined our sample size (see Appendix B in Supplemental materials), all data exclusions (if any), all manipulations, and all measures in the study, and we follow JARS [29]. The data necessary to reproduce the analyses presented here are not publicly accessible. Data can be made available upon contacting the corresponding author and under the conditions of a Data Use Agreement and pre-approval from an Institutional Review Board. Analytic code to reproduce the analyses in this paper are available from the first author. The parent study’s design was preregistered prior to data being collected, and the hypotheses for this specific study were preregistered after data were collected but before analyses were undertaken. The preregistration, including hypotheses and materials, are available through the Open Science Framework at https://osf.io/5q89s/?view_only=51cc38cf11fa4b3bba9f66c64ec812a8.

### Analysis Plan

Initial analyses using data from the pilot sample were conducted to identify an appropriate structure to represent the domains assessed by each of the four measures. These analyses then informed the selection of the final pool of items administered to the cross-validation sample. The first step in the analysis involved having the research team collaboratively develop potential models of the factor structure of each measure based on the themes and subdomains that guided the development of items included in the pilot survey. A confirmatory factor analysis (CFA) was then used to evaluate each proposed model using data from the pilot sample. Analyses were run in Mplus using maximum likelihood robust estimates to address missing data. Model fit was evaluated based on the following recommended cutoffs for the root mean square error of approximation (RMSEA), and comparative fit index (CFI). RMSEA ≤ 0.05 was indicative of close fit, RMSEA between 0.05 and 0.08 was indicative of fair fit, and values > 0.10 were indicative of poor fit between the hypothesized model and the observed data [30]. CFIs above 0.95 were considered to indicate a very good fit, and values between 0.90 and 0.95 generally considered acceptable. These were only general guidelines given the many factors that influence fit indices. In particular, measures of relative fit, such as the CFI, are typically lower when analyses are conducted on item-level indicators because they tend to have low intercorrelations [31]. In cases where none of the proposed models adequately fit the data, exploratory analyses were used to identify an alternative model. Details regarding the results of all analyses of the models tested for each measure are reported in appendices C to F in the Supplemental materials.

After a decision regarding the factor structure of each measure had been made, it was used to select the most appropriate set of items to include in the survey administered to the cross-validation sample. This involved excluding items that poorly represented the factors based on substantive considerations and factor loadings. Analyses of the cross-validation sample were then conducted to determine the extent to which the fit of the models identified from analyses of the pilot sample could be replicated in the larger, independent cross-validation sample. The cross-validation sample was then used to evaluate the fit of a comprehensive model that included items from all four measures. The cross-validation sample was also used to conduct tests of measurement invariance across sex and ethnicity. This involved multiple group analyses to compare the fit of increasingly restrictive models that assumed configural invariance (i.e., same factors and patterns of factor loadings across groups), metric invariance (i.e., same factor loadings across groups), and scalar invariance (i.e., same factor loadings and item intercepts across groups). Finally, analyses of covariance (ANCOVAs) on data from the cross-validation sample tested hypotheses regarding how scores on the measures of firearm-related attitudes and beliefs differed across sex and ethnicity, and participants who differed on measures of firearm-related practices, exposures, and influences. Each model included sex and ethnicity as covariates. These analyses were conducted on manifest variables created by averaging responses across items within each of the subscales. This method of scoring is typically used for most measures and it was assumed that future researchers were more likely to use this approach. Sensitivity analyses to determine if running ANCOVAs that treated the beliefs and attitude measures as latent variables rather than as manifest variables would have resulted in a different pattern of findings (see Appendix G in Supplemental materials for details).

## Results

### Initial Analyses of the Structure of Each Measure

Analyses of the 12-item General Attitudes Toward Firearms Measure administered to the pilot sample evaluated a 1-factor baseline model, and three models proposed by the research team. These included a 3-factor 12-item model, a 2-factor 11-item model, and a 2-factor 9-item model. A review of fit indices and factor loadings from CFAs based on each of these models led to the identification of a 2-factor model with separate factors representing Positive Attitudes Toward Firearms (5 items) and Negative Attitudes Toward Firearms (2 items) (see Appendix C in Supplemental materials for details). Based on these findings, an 8-item version of the measure that included an additional item representing negative attitudes was administered to the cross-validation sample. A 1-factor model based on these eight items fit the data very well, (χ2(20) = 129.23, RMSEA=0.06, CFI=0.95; all loadings were good to excellent (|λ| = 0.58 to 0.83). Although a 2-factor model had a better overall fit (χ2(19) = 64.18, RMSEA=0.04, CFI=0.98), the high negative correlation between the two factors (*r* = − .90) suggested they did not represent distinct constructs. On this basis we decided to focus on the 1-factor model based on its excellent fit and the limited practical value of creating separate factors that were so highly correlated.

CFAs of the 21-item Beliefs About Defensive Firearm Use Measure included in the pilot survey evaluated a 1-factor baseline model, and 2-factor, 3-factor, and 4-factor models proposed by the research team. None of these models adequately fit the data (see Appendix D in Supplemental materials for details). Exploratory factor analyses of data from the pilot sample led to the identification of a 3-factor 10-item version of the measure with factors representing Compensatory Force, Respect Enforcement, and Defensive Escalation. This model had an acceptable fit in both the pilot sample (χ2(32) = 166.01, RMSEA=0.088, CFI=0.90), and the cross-validation sample (χ2(32) = 254.07, RMSEA=0.065, CFI=0.92).

CFAs of the 9-item Prescriptive Norms for Threat Response Measure administered to the pilot sample evaluated a baseline 1-factor model, and three models specifying two or three factors proposed by the research team. Among the four models, a 2-factor model that specified factors representing Prescriptive Norms for Defense and Protection, and Prescriptive Norms for Reputation and Respect, was the most promising (see Appendix E in Supplemental materials for details). This model had an acceptable fit (χ2(26) = 155.32, RMSEA=0.089, CFI=0.90). Excluding three items with the lowest loadings improved the fit in for the pilot sample (χ2(8) = 42.88, RMSEA=0.089, CFI=0.95), and also had an acceptable fit in the cross-validation sample (χ2(8) = 118.36, RMSEA=0.091, CFI=0.93).

CFAs of the 20-item Attitudes Toward Firearm-Related Policy Measure administered to the pilot sample indicated that neither a baseline 1-factor model nor a 4-factor model proposed by the research team adequately fit the data (see Appendix F in Supplemental materials for details). A follow-up exploratory factor analysis of the pilot survey data suggested two factors representing Negative Attitudes Toward General Firearm Policies, and Positive Attitudes Toward Specific Firearm. A follow-up CFA based on these factors was conducted on a model that excluded four items that had poor loadings on both factors and four items based on substantive considerations. This revised 2-factor model had an acceptable fit in the pilot sample (χ2(53) = 225.36, RMSEA=0.077, CFI=0.93), and also fit the data well for the cross-validation sample (χ2(53) = 394.12, RMSEA=0.062, CFI=0.95).

### Confirmatory Factor Analysis of Items Across All Four Measures

After the separate analyses to identify the structure of each measure had been completed, a CFA of data from the nationally representative cross-validation sample was conducted to evaluate a comprehensive model that included all 36 items representing the eight factors across the four measures. This overall model had an excellent fit based on the RMSEA and an acceptable fit based on the CFI (χ^2^[566] = 2231.49, RMSEA = 0.04, CFI = 0.90). The items included within each factor and their standardized factor loadings are reported in Table 1. Based on suggested guidelines, all but one of the 36 standardized loadings were at least good (i.e., λ ≥ 0.55), 7 were very good (i.e., λ ≥ 0.63), and 23 were excellent (i.e., λ ≥ 0.71) [32]. Reliability coefficients (omega coefficients) ranged from 0.65 to 0.92; all but two were 0.80 or higher (see Table 2). The 28 correlations among the eight factors ranged in absolute value from 0.07 to 0.73, with a median value of 0.30 (see Table 2). This overall pattern suggests that the eight factors represent related but distinct constructs. Six of the correlations exceeded 0.50 in absolute value, but none were larger than 0.73. Nine correlations had absolute values between 0.30 and 0.50. The remaining 13 were between − 0.30 and 0.30. As expected, the Positive Attitudes Toward Specific Firearm Policies factor was negatively correlated with the other seven factors (*r*s = − 0.62 to − 0.18), which represented favorable attitudes toward firearms. Except for one small negative correlation (*r* = − .07), all correlations among the other seven factors were positive as expected. Table 2 also reports the means, standard deviations, and correlations among subscale scores calculated by taking the average rating across items within each scale. The overall pattern of correlations among subscale scores was similar to the pattern found for latent variables. Internal consistency based on alpha coefficients for subscale scores ranged from 0.62 to 0.91; all but one were above 0.75.

**Table 1:** Standardized factor loadings and reliabilities for confirmatory factor analysis of all four attitude measures

Measure and Items	Loading	SE
*General attitudes toward firearms*		
Firearms are necessary	0.81	0.015
I have the right to own a firearm	0.66	0.034
Firearms are the best way to protect yourself	0.80	0.015
Firearms are tools	0.57	0.027
Firearms don’t kill people. People kill people	0.75	0.015
Firearms do more harm than good	-0.83	0.020
Firearms are scary	-0.69	0.021
Firearms are bad	-0.82	0.014
*Beliefs about defensive firearm use*		
*Respect enforcement factor*		
If someone disrespects a female family member, then it is okay to shoot at them	0.78	0.025
If you find out your partner is cheating on you, it’s okay to go get a firearm	0.87	0.023
If your partner tries to leave you, it’s okay to point a firearm at them	0.84	0.024
If someone killed a person who you’re close to, it’s okay to go kill them	0.56	0.031
*Defensive escalation factor*		
If you see someone getting attacked, it’s okay to shoot the attacker	0.69	0.027
If someone points a firearm at you, it’s okay to shoot them	0.78	0.020
If someone breaks into your house, it’s okay to go get a firearm	0.80	0.024
*Compensatory force factor*		
If someone makes you feel threatened, it’s okay to tell them you have a firearm	0.72	0.025
If someone flashed a firearm at you, it’s okay to go get your firearm and bring it back	0.72	0.023
People will not mess with you if they know you have a firearm	0.39	0.037
*Prescriptive norms for threat response*		
*Defense and protection factor*		
People should be ready to defend themselves	0.90	0.015
People should be prepared to defend their homes	0.93	0.016
Men should protect the women in their lives	0.56	0.033
*Reputation and respect factor*		
People need to keep up a reputation to stop others from messing with them	0.73	0.026
People should always stand their ground	0.76	0.029
When people get disrespected, they need to do something about it	0.72	0.026
*Attitudes toward firearm-related policy*		
*Negative attitudes toward general firearm policies factor*		
Firearm control laws go against my Second Amendment rights	0.91	0.012
Firearm control laws hurt law-abiding citizens	0.92	0.014
Firearm control laws could prevent firearm violence, like…	-0.62	0.032
Firearm laws would not work because firearms are already in circulation	0.66	0.023
*Positive Attitudes toward specific firearm policies factor*		
No one should own AR-15-style semiautomatic rifles	0.70	0.021
People should be required to get firearm training before they can purchase firearm	0.70	0.022
Preventing people who have committed violent crimes from owning or using firearms	0.56	0.037
Raising the minimum age to buy any kind of firearm from 18 to 21	0.77	0.018
Requiring a permit for concealed carry	0.84	0.016
Requiring universal background checks for all firearm sales	0.86	0.015
Requiring a waiting period of 3 to 5 days before someone can buy a firearm	0.88	0.012
Laws that allow law enforcement officers to remove firearms from dangerous people	0.78	0.020

Analyses are based on the cross-validation sample (*N* = 1,675). Model fit: *χ*^*2*^(566) = 2231.49, RMSEA = 0.04, CFI = 0.90.

**Table 2:** Intercorrelations among latent factors (above diagonal) and subscale scores (below diagonal)

Subscale/Factor	1	2	3	4	5	6	7	8	Omega
1. General attitudes toward firearms	1.00	0.07	0.52	0.33	0.55	0.22	0.73	− 0.59	0.91
2. Beliefs supporting respect enforcement	0.10	1.00	0.08	0.46	− 0.07	0.29	0.14	− 0.26	0.85
3. Beliefs supporting defensive escalation	0.44	0.20	1.00	0.68	0.41	0.19	0.4	− 0.25	0.80
4. Beliefs supporting compensatory force	0.32	0.41	0.53	1.00	0.18	0.40	0.31	− 0.25	0.65
5. Prescriptive norms for defense and protection	0.49	0.01	0.35	0.23	1.00	0.41	0.40	− 0.25	0.85
6. Prescriptive norms for reputation and respect	0.19	0.24	0.14	0.29	0.39	1.00	0.30	− 0.18	0.78
7. Negative attitudes toward general firearm policies	0.71	0.16	0.35	0.29	0.40	0.23	1.00	− 0.62	0.87
8. Positive attitudes toward specific firearm policies	− 0.59	− 0.29	− 0.27	− 0.27	− 0.28	− 0.16	− 0.61	1.00	0.92
Descriptive statistics for subscale scores									
Mean	3.31	1.45	3.21	2.45	3.96	2.8	2.8	4.09	
Standard deviation	0.93	0.6	0.95	0.82	0.75	0.85	1.09	0.87	
Coefficient alpha	0.91	0.84	0.80	0.62	0.82	0.77	0.86	0.91	

Values based on the cross-validation sample. All correlations above 0.05 are significant at *p* < .05. Scale scores were calculated by taking the mean across items within each subscale. Omega coefficients represent reliability (internal consistency) of latent variables. Alpha coefficients represent reliability for subscale scores.

### Measurement Invariance Across Sex and Ethnicity

Measurement invariance across sex and ethnicity was evaluated using multiple group analyses of the data from the cross-validation sample. The multiple group model for sex was based on 867 women and 794 men. The multiple group model for ethnicity was limited to the three largest groups (i.e., *N* = 202 Black participants, 303 Latine participants, and 1,028 White participants). Because χ^2^ difference tests comparing the fit of models in large samples are overly sensitive to small, unimportant deviations from a perfect fit, we followed Chen’s [33] recommendations that measurement invariance was not supported if it (a) resulted in a poorer fit on the CFI (ΔCFI ≤ − 0.01) *and* (b) reduced the fit on either the RMSEA (ΔRMSEA ≥ 0.02) or on the standardized root mean square residual (ΔSRMR ≥ 0.03 for testing metric invariance and ΔSRMR ≥ 0.01 for tests of scalar invariance) [37]. None of the decreases in fit for comparisons of the metric invariance model to the configural invariance model, or comparisons of the scalar invariance model to the metric invariance model, exceeded the cutoffs for the RMSEA, SRMR, or CFI with one exception (see Table 3). Although the scalar invariance model for ethnicity decreased the CFI by 0.01, it did not meet the additional criterion of reducing either the RMSEA by 0.02 or more or the SRMR by 0.10 or more. Based on these findings, we concluded that consistent with hypotheses, the factor structures were consistent across ethnicity and gender.

**Table 3:** Fit indices for tests of measurement invariance across sex and ethnicity

Model		χ^2^	df	RMSEA	SRMR	CFI	ΔRMSEA	ΔSRMR	ΔCFI	
Multiple group by sex^a^										
Configural invariance	3022.46		1132	0.045	0.070	0.885				
Metric invariance	3107.54		1160	0.045	0.073	0.893	0.000	0.003	0.008	
Scalar invariance	3250.06		1188	0.048	0.074	0.887	0.003	0.001	− 0.006	
Multiple group by ethnicity^b^										
Configural invariance	4400.87		1698	0.056	0.075	0.869				
Metric invariance	4478.47		1754	0.055	0.081	0.868	− 0.001	0.006	− 0.001	
Scalar invariance	4769.04		1810	0.059	0.085	0.856	0.004	0.004	− 0.012	

Values based on analyses of the cross-validation sample. *χ*^*2*^ represents overall model fit. RMSEA = root mean square error of approximation. SRMR = standardized room mean square residual. CFI = comparative fit index. ΔRMSEA = change in RMSEA relative to less restrictive model. ΔSRMR = change in SRMR relative to less restrictive model. ΔCFI = change in CFI relative to less restrictive model.

^a^Groups consisted of 867 women and 794 men.

^b^Groups consisted of 1028 non-Hispanic White individuals, 303 Latine individuals, and 202 non-Hispanic Black individuals.

### Sex and Ethnic Differences in Beliefs and Attitudes

A series of 2 (sex) x 5 (ethnicity) ANCOVAs of data from the cross-validation sample investigated subscale differences associated with sex and ethnicity. Effect size estimates (d-coefficients) representing mean differences in subscale scores (manifest variables) are reported in Table 4. Significant sex differences were found for six of the eight subscales. As hypothesized, men had significantly higher scores for Positive Attitudes Toward Firearms, Respect Enforcement, Prescriptive Norms for Defense and Protection, and Negative Attitudes Toward General Firearm Policies (*d*s = 0.15 to 0.32), and lower scores for Positive Attitudes Toward Specific Firearm Policies (*d* = 0.46). Contrary to hypotheses, there were no differences between men and women on Prescriptive Norms for Reputation and Respect. Exploratory analyses of sex differences in Defensive Escalation indicated that men had higher scores then women (*d* = 0.32).

**Table 4:** Standardized differences in subscale means (d-coefficients) on attitude measures by sex and by ethnicity

Measure and subscale	Sex^a^	Ethnicity^b^		
	Men/ Women	Black/ White	Black/ Latine	White/ Latine
*General Attitudes Toward Firearms*				
Positive Attitudes Toward Firearms	0.30***	-0.38***	-0.11	0.27***
*Beliefs About Defensive Firearm Use*				
Respect Enforcement	0.15*	0.73***	0.32*	-0.40***
Defensive Escalation	0.32***	-0.03	0.07	0.10
Compensatory Force	0.03	0.17	0.06	-0.11
*Prescriptive Norms for Threat Response*				
Defense and Protection	0.22***	0.10	0.18	0.08
Reputation and Respect	-0.09	0.24*	0.03	-0.21*
*Attitudes Toward Firearm-Related Policy*				
Negative Attitudes Toward General Firearm Policies	0.15*	-0.18	-0.24*	-0.06
Positive Attitudes Toward Specific Firearm Policies	-0.46***	-0.04	0.01	0.05

Analyses are based on the cross-validation sample. Coefficients are the standardized difference between the mean in first group listed minus the mean in second group listed in each column heading.

^a^*N* = 867 women and 794 men. ^b^*N* = 1,039 non-Hispanic White, 298 Latinx, 202 non-Hispanic Black participants. The analysis sample included 67 non-Hispanic Asian and 55 non-Hispanic multiracial participants, but comparisons with these groups are not reported due to the small number of participants within these groups.

*p < .05. **p < .01. ***p < .001.

Most analyses of ethnic differences were considered exploratory. Although the analyses included participants from all five categories of ethnicity, pairwise comparisons were only examined across the three groups best represented in the sample. As hypothesized, White participants reported more favorable attitudes on the Positive Attitudes Toward Firearms scale than both Black and Latine participants (*d*s = 0.38 and 0.27, respectively); and Black participants had stronger beliefs supporting use of firearms to enforce respect than did White participants (*d* = 0.73). Contrary to hypotheses, there were no differences between Black and White adults on the two subscales assessing Attitudes Toward Firearm-Related Policy. Exploratory analyses indicated that compared with Latine participants, Black participants endorsed beliefs supporting firearm use more positively (*d* = 0.32) and White participants endorsed them less positively (*d* =- 0.40). In addition, Black and Latine participants endorsed beliefs more supportive of Prescriptive Norms for Reputation and Respect than did White participants (*d*s = 0.24 and 0.21, respectively). Finally, Latine participants reported more Negative Attitudes Toward General Firearm Policies than Black participants (*d* = 0.24). Sensitivity analyses conducted on latent variables revealed the same pattern of significant effects across sex and ethnicity that were found in the analyses of manifest variables with one exception – sex differences were not found for Respect Enforcement (see Table G-1 in Supplemental materials).

### Associations Between Belief and Attitude Measures and Firearm-Related Variables

#### Associations with Firearm Access, Carrying, and Storage

Standardized effects from ANCOVAs investigating mean differences on the eight attitude subscales across subgroups that differed on firearm access, carrying, and storage practices are reported in Table 5. As hypothesized, participants who owned a firearm had significantly higher scores on all but two of the subscales that reflected supportive attitudes toward firearms and their use, and less positive attitudes toward specific firearm policies compared with participants without access to a firearm (absolute value of *d*s from 0.28 to 0.95). Although participants who owned a firearm also expressed more favorable attitudes toward firearms compared with those with household ownership, the differences were generally smaller (absolute value of *d*s from 0.27 to 0.50) than in comparisons with those having no access. Participants with household firearm ownership had higher scores on general attitudes, defensive escalation, and negative attitudes toward firearm-related policies (*d*s = 0.22 to 0.45), and lower scores on positive attitudes toward specific firearm policies (*d* = − 0.35) than participants with no access.

**Table 5:** Standardized differences in subscale means (d-coefficients) on attitude measures across groups differing in firearm-related access and practices

Measure and subscale	Firearm Access^a^				
	Personal Ownership/ No Access	Personal/ Household Ownership	Household Ownership / No Access	Carry: Yes/No^b^	Unsafe storage Yes/No^c^
*General Attitudes Toward Firearms*					
Positive Attitudes Toward Firearms	0.95***	0.50***	0.45***	0.82***	0.53***
*Beliefs About Defensive Firearm Use*					
Respect Enforcement	-0.03	-0.10	0.07	0.17	-0.08
Defensive Escalation	0.51***	0.29**	0.22*	0.52***	0.69***
Compensatory Force	0.28***	0.27**	0.01 -	0.38***	0.36**
*Prescriptive Norms for Threat Response*					
Defense and Protection	0.54***	0.39***	0.14	0.55***	0.54***
Reputation and Respect	0.00	0.09	-0.10	0.34**	0.20
*Attitudes Toward Firearm-Related Policy*					
Negative Attitudes Toward General Firearm Policies	0.64***	0.30**	0.34**	0.78***	0.60***
Positive Attitudes Toward Specific Firearm Policies	-0.65***	-0.29**	-0.35***	-0.83***	-0.53***

Analyses are based on the cross-validation sample. Coefficients are the standardized difference between the mean in first group listed minus the mean in second group listed in each column heading, controlling for sex and ethnicity.

^a^*N* = 505 firearm owners, 146 with access to a firearm in their home, and 1,010 who reported not owning or having access. ^b^*N*s = 178 firearm owners who reported that they carry a firearm, and 260 firearm owners who reported they do not carry a firearm. ^c^*N*s = 149 firearm owners who reported using unsafe storage practices, and 317 firearm owners who did not report unsafe storage practices.

*p < .05. **p < .01. ***p < .001.

Analyses of differences in attitudes across subgroups that differed in firearm carrying and storage practices were restricted to participants who reported owning a firearm. As hypothesized, firearm owners who reported carrying a gun had higher scores on all but one of the measures that assessed favorable attitudes toward firearms and had less positive attitudes toward specific firearm policies (*d* = − 0.83) compared with those who reported not carrying (*ds =* 0.34 to 0.82). Firearm owners who reported unsecured firearm storage practices also endorsed more favorable attitudes toward firearms and their use on all but two of the eight scales (absolute values of *ds =* 0.36 to 0.69) compared with those who reported using secure storage practices.

#### Associations with Firearm-Related Violence

Effect size estimates for ANCOVAs examining differences on attitude measures for subgroups who differed on firearm-related perpetration, victimization, and witnessing are reported in Table 6. Only two of the six hypotheses for relations between attitudes and firearm-related perpetration were supported. Participants who reported that they had used a firearm against someone reported more positive attitudes toward firearms, and more support for defensive escalation (*ds =* 62 and 0.36, respectively). Exploratory analyses of the Attitudes Toward Firearm-Related Policy measure indicated that participants who had used a firearm against someone endorsed more negative attitudes toward general firearm policies, and less positive attitudes toward specific policies (*ds =* 0.42 and − 0.66, respectively). Given the potentially confounding difference in rates of firearm-related perpetration related to owning or not owning a firearm (i.e., 11% and 3%, respectively), these relations were further tested in a model that controlled for firearm ownership (see Model 2 in Table 6). Within this model, relations between firearm-related perpetration and general attitudes toward firearms and positive attitudes toward specific policies remained significant, but decreased in magnitude, but the relation between firearm-related perpetration and subscales representing defensive escalation and negative attitudes toward general firearms policies did not.

**Table 6:** Standardized differences between subscale means (d-coefficients) on attitude measures across groups differing in firearm-related perpetration, victimization, and witnessing

Measure and subscale	Firearm-Related Perpetration: Yes/No^a^		Firearm-Related Victimization Yes/No^b^	Firearm-Related Witnessing Yes/No^c^
	Model 1	Model 2		
*General Attitudes Toward Firearms*				
Positive Attitudes Toward Firearms	0.62***	0.35***	0.14	0.24**
*Beliefs About Defensive Firearm Use*				
Respect Enforcement	0.29	0.30*	0.20**	0.07
Defensive Escalation	0.36***	0.21	0.15	0.29***
Compensatory Force	0.30	0.24	0.09	0.06
*Prescriptive Norms for Threat Response*				
Defense and Protection	0.23	0.07	0.04	-0.01
Reputation and Respect	0.14	0.15	0.09	0.13
*Attitudes Toward Firearm-Related Policy*				
Negative Attitudes Toward General Firearm Policies	0.42**	0.22	0.14	0.12
Positive Attitudes Toward Specific Firearm Policies	-0.66***	-0.45**	-0.20**	-0.13

Analyses are based on the cross-validation sample. Coefficients are the standardized difference between the mean in first group listed minus the mean in second group listed in each column heading, controlling for sex and ethnicity.

^a^*N* = 88 who reported using a firearm against someone and 1,568 who did not. Model 2 controlled for owning a firearm; Model 1 did not. ^b^*N* = 375 who reported being threatened, shot at, or injured by a firearm, and 1,286 who did not. ^c^*N* = 229 who reported seeing someone else get shot at, and 1,432 who did not.

*p < .05. **p < .01. ***p < .001.

Firearm-related victimization was associated with more favorable attitudes toward defensive firearm use to enforce respect and less positive attitudes toward specific policies (*d* = 0.20 in absolute value for both). The patterns of relations between witnessing violence and attitudes supported only one out of four hypotheses. Participants who witnessed firearm-related violence had more positive attitudes beliefs supporting firearm use for defensive escalation (*d* = 0.29). They also endorsed more positive general attitudes toward firearms (*d* = 0.24).

#### Associations with Family and Friend Influences

Effect size estimates for ANCOVAs examining relations between attitude measures and family and friend factors are reported in Table 7. All hypotheses related to the association between friends’ firearm carrying and attitudes were supported. Friends’ firearm carrying was associated with more supportive attitudes toward firearms and their use, and more negative attitudes toward firearm-related policies, across all eight subscales (see Table 7). These included small differences in respect enforcement, compensatory force, and reputation and respect (*ds =* 0.21 to 0.33); moderate differences in defensive escalation, norms for defense and protection, and positive attitudes toward specific firearm policies (*d*s = 0.42, − 0.56), and large differences in positive attitudes toward firearms and negative attitudes toward general policies (*ds =* 0.85 and 0.70, respectively). Given the significant relation between owning a firearm and friends’ firearm carrying (phi = 0.24, *p* < .001), we again analyzed a second model that controlled for firearm ownership (Model 2; Table 7). Although this resulted in a slight decrease in the effect sizes, all differences remained significant. Participants who reported having firearms in the home when they were growing up expressed more favorable attitudes toward firearms on all the subscales except for beliefs supporting the use of firearms for enforcement of respect. These were small to medium effects (absolute value of *ds =* 0.13 to 0.49), except for larger effects on positive attitudes toward firearms and negative attitudes toward general policies (*ds =* 0.66 and 0.53, respectively). A second model that controlled for both firearm ownership and having household firearm access (see Model 3 in Table 7) resulted in some reduction in effect sizes; all remained significant except for the small effect that had been found for compensatory force.

**Table 7:** Standardized differences in subscale means (d-coefficients) on attitude measures across groups differing in firearm-related friends and family influence variables

Measure and subscale	Friends carry firearm: Yes/No^a^		Firearms in home growing up: Yes/No^b^		Peer/family firearm injury: Yes/No^c^	Peer/family firearm suicide: Yes/No^d^
	Model 1	Model 2	Model 1	Model 3		
*General Attitudes Toward Firearms Measure*						
Positive Attitudes Toward Firearms	0.85***	0.69***	0.66***	0.46***	-0.04	0.31***
*Beliefs About Defensive Firearm Use*						
Respect Enforcement	0.21***	0.23***	0.12	0.15*	0.18*	0.09
Defensive Escalation	0.42***	0.34***	0.33***	0.22***	0.09	0.26***
Compensatory Force	0.33***	0.29***	0.16*	0.09	0.05	0.10
*Prescriptive Norms for Threat Response*						
Defense and Protection	0.56***	0.48***	0.35***	0.22***	0.04	0.23**
Reputation and Respect	0.32***	0.34***	0.13*	0.14*	0.09	0.19*
*Attitudes Toward Firearm-Related Policy*						
Negative Attitudes Toward General Firearm Policies	0.70***	0.60***	0.53***	0.41***	-0.03	0.18
Positive Attitudes Toward Specific Firearm Policies	-0.55***	-0.43***	-0.49***	-0.36***	0.08	-0.36***

Coefficients represent standardized difference between mean in first group minus second group listed in each column heading, controlling for sex and ethnicity.

^a^*N* = 1,090 who reported at least some friends carried firearms, and 558 who did not. Model 2 controlled for owning a firearm, Model 1 did not. ^b^*N* = 843 who reported having firearms in their home growing up, and 814 who reported they did not. Model 3 controlled for personal firearm ownership or household firearm ownership, Model 1 did not. ^c^*N* = 335 who reported having a friend or a family member injured or killed due to violence involving a firearm, and injured seeing someone else get shot at, and 1,326 who did not. ^d^*N* = 263 who reported having a friend or a family member attempt or commit suicide using a firearm, and 1,396 who did not.

*p < .05. **p < .01. ***p < .001.

Individuals who reported having a peer or family member experience a firearm-related injury or suicide were hypothesized to have a similar pattern across five of the belief and attitude subscales. None of these hypothesized effects were found for having a peer or family member experience a firearm-related injury. In contrast, hypothesized effects were found for participants who reported that a friend or family member had attempted or committed suicide. More specifically, they reported stronger beliefs supporting the use of firearms in defensive escalation, prescriptive norms for defense and protection, and prescriptive norms for reputation and respect (*d*s = 0.19 to 0.26). Counter to hypotheses participants who reported that a friend or family member had attempted or committed suicide endorsed less positive attitudes toward specific firearm policies (*d* = − 0.36). Exploratory analyses also indicated that they had more positive attitudes toward firearms (*d* = 0.31).

#### Sensitivity Analyses

Results of the sensitivity analyses of ANCOVAs based on latent variables rather than on manifest variables are reported in Tables G-2 to G-5 in Supplemental materials. Out of 74 effect size estimates that were significant at *p* < .05 in the analysis of manifest variables, all but three were also significant in the analysis based on latent variables. Values of these estimates differed by at most 0.13. All but three differed by less than 0.10 and most (73%) differed by 0.05 or less. 70% of the time estimates were higher for latent variables versus manifest variables.

## Discussion

The purpose of this study was to develop and test the validity of four community-informed measures designed to assess firearm-related attitudes and beliefs, and examine their associations with firearm-related practices and violence exposure. We identified factor structures for each measure (General Attitudes Toward Firearms, Beliefs About Defensive Firearm Use, Prescriptive Norms for Threat Response, and Attitudes Toward Firearm-Related Policy) based on the pilot data, that were cross validated in the larger independent sample. Support was also found for strong measurement invariance across ethnicity and sex. These measures are an important addition to the existing scales assessing firearm-related beliefs and attitudes. For example, the range of subscales identified points to the highly nuanced nature of these constructs. Rather than being unidimensional scales, even a construct as focused as attitudes about firearm-related policy resulted in two separate subscales (Negative Attitudes Toward General Firearm Policies and Positive Attitudes Toward Specific Firearm Policies) that had unique patterns of relations to demographic characteristics and involvement in firearm-related violence. Across the measures, subscales within these measures displayed unique patterns of relations with firearm-related practices and firearm-related family and friend influences and exposures. This highlights the potential value of these community-informed measures of firearm-related attitudes and beliefs for improving the understanding of firearm-related behaviors and violence exposure. Overall, these findings are consistent with social-cognitive theory [9], as evidenced by a general pattern of associations between individuals’ attitudes and beliefs and their firearm-related experiences and behaviors.

As hypothesized, scores on the measures of general positive attitudes towards firearms was higher among men and White adults. Consistent with prior research [8, 15], individuals who personally owned firearms or who had firearms in their homes also had more positive attitudes toward firearms. This study built on this research by providing novel information about the relation between generally positive attitudes and firearm-related experiences or practices. More specifically, individuals who carried firearms, who engaged in unsecured storage practices in their home, and who had perpetrated or witnessed firearm-related violence had more positive attitudes toward firearms, as did individuals whose friends carried firearms, who had a close family member or friend who had been injured or killed due to suicide, and who had firearms in their homes growing up. Based on social-cognitive theory, it is likely that exposure to close others’ engagement in firearms promotes positive attitudes toward firearms, and that one’s own behaviors (e.g., ownership, carrying, storage) are both driven by and reinforce those positive attitudes. It is notable that using a firearm against someone else and witnessing firearm-related violence were each associated with positive attitudes toward firearms even after controlling for firearm ownership. It is possible that these relations are reciprocal; for example, a person may believe in using firearms for self-defense and therefore use a firearm against someone else, which may then reinforce their positive attitudes about firearms. More research is needed to understand the directionality of the relations between these attitudes, experiences, and practices.

Subscales on the Beliefs About Defensive Firearm Use measure also had interesting patterns of relations to firearm-related practices and experiences. The constructs they represent may be closely related to normative beliefs about aggression—that is, beliefs about when aggression is an appropriate or effective response [34]. In general, beliefs supporting aggressive behavior are predicted by exposure to violence and predict aggression [35]. Beliefs supporting firearm use for respect enforcement were higher among Black and Latine adults compared to White adults, but unrelated to sex. Beliefs supporting firearm use for defensive escalation were higher among men but unrelated to ethnicity, and beliefs supporting firearm use for compensatory force were consistent across sex and ethnicity. Beliefs supporting respect enforcement were unrelated to firearm-related practices, but both beliefs supporting defensive escalation and compensatory force were related to all firearm-related practices in the expected directions, including firearm ownership, carrying, and unsecured storage. These findings are consistent with prior findings indicating that firearm owners are more likely to support the defensive use of firearms against intruders [8]. Again, it may be that these beliefs drive firearm-related practices (e.g., individuals who strongly believe in using firearms when threatened or during a home invasion may be more likely to carry a firearm and store it in an easily accessible manner at home). It is also possible that these practices reinforce these beliefs. Beliefs supporting defensive escalation were higher among individuals who had used a firearm against someone else or who had witnessed firearm-related violence, but neither beliefs supporting respect enforcement nor compensatory force were related to violence involvement. Beliefs supporting defensive escalation may be particularly important in understanding the cycle of firearm-related violence, such that witnessing violence may lead to an increased perceived need to defend oneself proactively, which may then result in the use of firearms against others. All beliefs supporting firearm use had higher endorsement among those whose friends carried firearms, suggesting that social group norms may influence these beliefs. Beliefs supporting defensive escalation were also higher among those whose family members or close friends had committed suicide and among those who had firearms in the home growing up. These patterns of relations may be related to firearm availability in one’s social circle and one’s degree of embeddedness in firearm culture.

The measures developed to assess prescriptive norms for responding to threats—norms for defense and protection, and norms for reputation and respect—are most closely related to beliefs labeled “code of the street,” “culture of honor,” or “masculine honor norms” in other work. This prior work has linked these patterns of beliefs to violent behavior (not specific to firearm use) and firearm ownership [20, 22]. This measure was different than the other measures developed for this study in that it did not specifically reference firearms, but was designed to assess underlying social-cultural norms that may underlie firearm-related behaviors and experiences. In addition, rather than asking about personal beliefs, the measure asks about what the people *around* the respondent believe, tapping into perceptions of social norms. As expected, norms for defense and protection were higher among men, but unexpectedly, norms for reputation and respect were comparable across sex. There were no differences in norms for defense and protection across ethnicity, but norms for reputation and respect were higher among Black and Latine adults than among White adults. In general, norms for defense and protection were associated with all firearm-related practices, including firearm ownership. Among firearm owners, these norms were stronger for those who carried and who used unsecured storage. Norms for reputation and respect were associated with higher carrying but no other firearm-related practices. Neither type of prescriptive norms for threat response were related to any type of firearm-related violence involvement, but they were both related to multiple family and friend factors, including friends’ carrying, friends’ and family members’ injury or death due to suicide, and growing up with firearms in the home. These results suggest that prescriptive norms toward threat response may be indicative of cultural factors linked to firearm availability and culture, but unrelated to actual involvement in firearm-related violence.

The Attitudes Toward Firearm-Related Policies measures generally showed the expected patterns of relations to demographic factors, firearm-related practices, and firearm-related exposures and influences. Of note, the average rating for attitudes toward specific policies was between “support” and “strongly support,” and the average rating for negative attitudes toward general firearm policy (ineffectiveness and harm) was between “neutral” and “disagree.” Consistent with hypotheses and prior research, men had more negative attitudes toward general and less support for specific firearm-related restrictive policies than women [11]. Unexpectedly, and inconsistent with other research, there were no differences between Black adults and White adults in negative general attitudes or specific positive attitudes toward firearm-related policies, although Latine adults had more negative attitudes toward general firearm policy than Black adults. Prior research on firearm-related policy attitudes has focused on individual policies and generally assessed support or opposition to those policies, suggesting that those results may be limited to the specific items being assessed, whereas this measure may assess a latent factor that taps into individuals’ broader stances toward firearm-related policies.

Both negative attitudes toward general firearm-related policies and positive attitudes toward specific firearm-related policies were associated with all firearm practices (levels of ownership, carrying, and unsecured storage) in the expected directions. This is consistent with prior research that has found less support for firearm-related policies among firearm owners [10]. Novel findings in the current study include that greater negative attitudes toward general firearm policies and lower positive attitudes toward specific firearm policies were associated with the use of firearms against someone else. Similarly, individuals who had experienced firearm-related victimization had fewer positive attitudes toward specific firearm-related policies. It is possible that individuals who have been involved in firearm-related violence may be less likely to believe in the efficacy and value of firearm-related policies, which they may believe either failed to protect them or may constrain their ability to respond effectively to perceived threats. Attitudes toward firearm-related policies were also related to family and friend influences; specifically, both types of attitudes were related to friends’ carrying practices and having firearms in the home growing up, in the expected directions. Furthermore, endorsement of positive attitudes toward specific firearm-related policies was lower among those whose family members or friends had been injured by firearms or died by suicide. As previously stated, these relations might be spurious, potentially caused by firearm availability in one’s social network, which increases the perceived importance of firearms in daily life, and would likely decrease agreement with externally imposed restrictions.

### Limitations

This study had several limitations that should be considered. Because it was a cross-sectional study, we cannot determine whether these attitudes and beliefs lead to behavioral choices, if behavioral choices lead to these attitudes and behaviors, or if other phenomena cause both. As this was not an experimental study, we cannot determine whether changing these attitudes and beliefs would change behaviors, or vice versa. Although this was a U.S. nationally representative sample, we did not have large enough samples of participants who identified as Asian or Asian American or American Indian or Alaska Native to examine whether these measures functioned the same for members of those groups as for White, Black, and Latine participants. In addition, we did not examine whether the patterns of relations between these firearm-related beliefs and attitudes with firearm-related experiences, practices, and influences varied across demographic groups, such as ethnicity, sex or gender, or region of the U.S. Future research examining these relations within groups could provide more nuance in the understanding of patterns of relations. Finally, as all the measures in this study were self-report, monomethod bias could potentially have inflated the size of the relations among constructs.

### Future Directions and Prevention Implications

The firearm-related measures developed in this study are an important step that could improve the understanding of the social-cultural context of firearm-related practices and behaviors and identify potential targets for intervention to prevent firearm-related violence involvement. Importantly, these measures were developed as part of a collaboration with individuals who own and use firearms, expanding the range of attitudes and beliefs that have been typically assessed in the literature, as well as potentially addressing bias in existing items. The findings emphasize the value of nuanced, multidimensional scales that capture a range of attitudes and beliefs related to firearms and their use.

There are several ways these measures can be used in future research to further our understanding of firearm-related violence and develop targeted interventions. Future work examining the validity of these measures for predicting risky firearm-related behaviors or firearm-related injury or harm in longitudinal designs, as well as exploring how these beliefs and attitudes develop over time, could provide important insights about how firearm-related experiences are connected to firearm practices. Research related to the development of negative attitudes toward general restrictive firearm policies in men, for instance, may help identify potential targets for reducing men’s negative attitudes and increasing their positive attitudes toward policies. Longitudinal research should examine firearm-related beliefs (e.g., beliefs about defensive firearm use and general attitudes) as mediators of relations between firearm-related violence exposure (e.g., witnessing, perpetration, and victimization) and firearm-related behaviors (e.g., ownership, carrying, and use). Given our findings related to friend and family influences (e.g., friends’ carrying), these measures may also be used to explore within group variability of firearm-related beliefs and behaviors in social networks and in families, as well as to explore relations between friend or family members’ firearm-related beliefs and individuals’ firearm-related beliefs and behaviors.

## Supplementary Information

Below is the link to the electronic supplementary material.

Supplementary Material 1

## Data Availability

This study and its hypotheses were preregistered, and materials are available at https://osf.io/hucbj. All data are available upon reasonable request from the corresponding author following a Data Use Agreement and IRB approval.
