# Supplementary material for: Community-Based Development of Tools to Assess Firearm-Related Attitudes and Beliefs: Associations with Firearm-Related Practices, Social Influences, and Violence Exposure
Source: J Community Health. 2026 Jun 5;51(5):730–45. doi: 10.1007/s10900-026-01587-6 (PMC13480196; doi:10.1007/s10900-026-01587-6)
Supplement: Supplementary file 1 [file 10900_2026_1587_MOESM1_ESM.docx]

**Supplemental Materials Appendix A - G**

**Title:** Community-Based Development of Tools to Assess Firearm-Related Attitudes and Beliefs: Associations with Firearm-Related Practices, Social Influences, and Violence Exposure

**Journal:** *Journal of Community Health*

**Authors:** Krista R. Mehari,^1^ Albert D. Farrell,^2^ Jasmine N. Coleman,^3^ Lindiwe S. Mayinja,^4^ Colleen S. Walsh,^5^ & Phillip N. Smith^6^

^1^Corresponding author. Vanderbilt University, Peabody College of Education and Human Development, Department of Psychology and Human Development. Email: [krista.mehari@vanderbilt.edu](mailto:krista.mehari@vanderbilt.edu).

^2^Virginia Commonwealth University, Department of Psychology.

^3^University of Tennessee-Knoxville, Department of Psychology.

^4^Vanderbilt University, Peabody College of Education and Human Development, Department of Psychology and Human Development,

^5^Vanderbilt University, Peabody College of Education and Human Development, Department of Psychology and Human Development.

^6^University of South Alabama, Department of Psychology.

**Table of Contents**

[**Appendix A**	3](#_Toc228110975)

[Preregistered Study Hypotheses	3](#_Toc228110976)

[**Appendix B**	5](#_Toc228110977)

[Sample Size and Power Analysis	5](#_Toc228110978)

[**Appendix C**	6](#_Toc228110979)

[Evaluation of General Attitudes Toward Firearms Measure	6](#_Toc228110980)

[**Appendix D**	12](#_Toc228110981)

[Evaluation of Beliefs About Defensive Firearm Use Measure	12](#_Toc228110982)

[**Appendix E**	22](#_Toc228110983)

[Evaluation of Prescriptive Norms for Threat Response Measure	22](#_Toc228110984)

[**Appendix F**	26](#_Toc228110985)

[Evaluation of Attitudes Toward Firearm-Related Policy Measure	26](#_Toc228110986)

[**Appendix G**	33](#_Toc228110987)

[Sensitivity Analysis Comparing ANCOVA Findings Using Manifest Variables versus Latent Variables	33](#_Toc228110988)

# **Appendix A**

## Preregistered Study Hypotheses

**Hypothesis 1.** The factor structures of the measures would be invariant across ethnicity and sex.

**Hypothesis 2.** The measures would have adequate divergent validity as indicated by small to moderate correlations with each other.

**Hypothesis 3.** The measures would have specific patterns of associations with demographic factors. Specifically: **(a)** Men and White adults would have more positive attitudes toward firearms. **(b)** Men and Black adults would more strongly endorse beliefs supporting firearm use to enforce respect. **(c)** Men would endorse greater prescriptive norms for defense and protection and greater prescriptive norms for reputation and respect. (**d)** Men and White adults would endorse more negative attitudes toward general firearm policy. Women and White adults would endorse greater positive attitudes toward specific firearm policy.

**Hypothesis 4.** The measures would have specific patterns of associations with firearm-related behaviors, practices, and violence or injury involvement. Specifically: **(a)** Positive attitudes toward firearms would be higher among firearm owners and individuals with household firearm access; individuals involved in firearm violence through perpetration or direct victimization; individuals with a greater proportion of friends who carry firearms; and individuals who grew up with firearms in the home. **(b)** Beliefs supporting firearm use to enforce respect would be higher among individuals who had been involved in firearm violence as perpetrators. **(c)** Beliefs supporting firearm use for defensive escalation would be higher among individuals who had been involved in firearm violence as perpetrators, victims, or witnesses; among individuals with a greater proportion of friends who carry firearms; and among individuals whose friends or family members had been injured or killed due to violence. **(d)** Prescriptive norms about defense and protection would be higher among firearm owners and individuals with household firearm access compared to those with no ownership/access; among individuals who carry firearms; among individuals who had been involved in firearm violence as perpetrators, victims, or witnesses; among individuals with a greater proportion of friends who carry firearms; among individuals who grew up with firearms in the home; and among individuals whose friends or family members had been injured or killed due to violence. **(e)** Prescriptive norms for reputation and respect would be higher among individuals who carry firearms; among individuals who have been involved in firearm violence as perpetrators, victims, or witnesses; among individuals whose friends carry firearms; and among individuals whose friends or family members had been injured or killed due to violence. **(f)** Negative attitudes toward general firearm policies would be higher among firearm owners and individuals with household firearm access compared to individuals without access; among individuals who carry firearms; among individuals whose friends carried firearms; and among individuals who grew up with firearms in the home; and would be lower among individuals whose families or friends were injured or killed due to suicide. **(g)** Positive attitudes toward specific firearm policies would be lower among firearm owners and individuals with household firearm access; among individuals who carry firearms; among individuals whose friends carry firearms; and among individuals who grew up with firearms in the home. These attitudes would be higher among individuals whose family members or friends were injured or killed due to violence or injured or killed due to suicide.

# **Appendix B**

## Sample Size and Power Analysis

Sample size for analyses of the structure of the measure was determined by *a priori* power analyses for testing measurement models in the pilot and cross-validation samples, using recommended procedures and software [31, 32]. For an estimated pilot sample of 500 adults, we obtained power estimates of >.99 for tests of close fit for models specifying between one and three factors based on measures ranging from 20 to 30 items (*df*s = 167 to 405). Power estimates for comparing the fit of a three-factor versus a one-factor model also exceeded .99 for detecting a difference between two close-fitting models (i.e., RMSEA = .05 versus .06). We used a similar approach to evaluate the analysis of the full measurement model based on an estimated 1700 adults in the cross-validation sample. We used an example for the full measurement model that assumed a total of 160 items representing 20 latent variables (*df* = 12,180). Our analysis provided estimates of power > .99 based on the smallest sample of an estimated 731 adults in households with firearms. We also obtained power estimates exceeding .99 for detecting differences between RMSEAs of .05 and .06 in comparisons of this model to a model that specified 5 higher-order social-ecological factors (dfs = 12,500). We examined power for tests of measurement invariance (e.g., comparing parameters for households with and without firearms). We again found power estimates > .99 to detect differences in RMSEAs between .05 and .06 for comparisons of constrained and unconstrained versions of the above models (*df*s ranged from 24,360 to 25,060).

# **Appendix C**

## Evaluation of General Attitudes Toward Firearms Measure

***Models Tested***

Confirmatory Factor Analyses (CFA) of data from the pilot sample evaluated a 1-factor baseline model (AAG-P1) and three models proposed by the research team. These included a 3-factor model that included all 12 items, a 2-factor 11-item model, and a 2-factor 9-item model. Fit indices for each of these models are reported in Table C-1 and the standardized loadings for each of these models are reported in tables C-2 to C-5. Model fit was evaluated based on the root mean square error of approximation (RMSEA), and comparative fit index (CFI). We used the following recommended fit indicators: RMSEA ≤ .05 was indicative of close fit, RMSEA between .05 and .08 was indicative of fair fit, and values > .10 were indicative of poor fit between the hypothesized model and the observed data [33]. Values for the CFI above .95 were considered to indicate a very good fit, with values for the CFI between .90 and .95 generally considered acceptable. However, it has long been noted that these cutoffs are general guidelines given the many factors that influence fit indices. For example, measures of relative fit, such as the CFI, are typically lower when analyses are conducted on item-level indicators because they tend to have lower intercorrelations [34].

The 1-factor model did not adequately fit the data. The 3-factor model (AAG-P2) resulted in an improper solution (i.e., one or more estimated correlation between two factors exceeded 1.00). The two 2-factor models (AAG-P3 and AAG-P4) appeared the most promising. Of the two, we focused on Model AAG-P3, because the two resulting factors were more independent than in Model AAG-P4 (i.e., *r* = -.79 versus .89), and because the interpretation of the two factors (i.e., Positive Attitudes Toward Firearms and Negative Attitudes Toward Firearms) appeared more straightforward than the alternative 2-factor model (i.e., Firearms are Useful, Firearms are a Problem).

Further review of the items in Model AAG-P3 revealed three items that their content did not clearly represent the two factors. Item 8 (Firearms are weapons) did not clearly represent a positive or negative attitude and had a poor loading on the Negative Attitudes Factor. Item 10 (Everyone should own a firearm) and Item 11 (No one should own a firearm) reflected extreme statements that few participants agreed with (11% and 15%, respectively). Item 12 (Everyone should be able to buy a firearm) also represented an extreme view and had a poor loading on the Positive Attitudes factor (see Table C-4). Based on these considerations, a modified 7-item version of Model AAG-P3 that excluded these four items was evaluated. This model fit the data very well (see Model AAG-P3R in Table 1) and all the standardized loadings ranged from good to excellent (see Table C-6). Reliability coefficients (omega) for the Positive Attitudes Toward Firearms and Negative Attitudes Toward Firearms were .86 and .82, respectively. Although the high magnitude of the correlation between the two factors in this model (*r* = -.76) suggested that the items might be represented by a single factor, a 1-factor model based on these seven items (see Table C-7) did not adequately fit the data (see Table C-1). The 2-factor 9-item model was considered the most promising model based on the pilot sample.

***Final Model***

The cross-validation sample was administered a shortened version of the General Attitudes Toward Firearms measure that excluded the items not included in the final model identified in the analyses of the pilot sample. An additional item (AAG13 Firearms are bad) was added to the survey to increase the number of items on the Negative Attitudes Toward Firearms factor. This resulted in a modified 8-item version of Model AAG-P3R that included the additional item. This model fits the data very well (see Table C-1). All standardized loadings were good to excellent (see Table C-6). As in the analyses of the pilot sample, there was a high negative correlation between the two factors (*r* = -.90). This suggested that the two sets of items may have had cross-loadings or represented a single factor. We ran an ESEM based on Model AAG-F3R to evaluate this. The absolute values of cross-loadings were all less than .20, with two exceptions (-.29 for Item AAG07 on the Positive Attitudes Toward Firearms factor and -.23 for item AAG04 on the Negative Attitudes Toward Firearms factor). This suggested that cross loadings did not account for the high correlation between the factors. A one-factor model based on the eight items fit the data very well, though not as well as the 2-factor model (see Model AAG-F1R in Table C-1). Although the 2-factor model had a better overall fit, we ultimately decided to focus on the 1-factor model because there seemed to be limited practical value in creating separate factors that had such a high correlation. All loadings for this factor were good to excellent (see Table C-7). The reliability (omega) for the General Attitudes Toward Firearms factor was .91.

**Table C-1**

*Fit Indices for Competing Models of the Structure of the Attitudes Toward Firearm-Related Policies Measure*

| Model | Description | χ2 | *df* | RMSEA | CFI |
| --- | --- | --- | --- | --- | --- |
| Analyses of pilot sample | | | | | |
| AAG-P1 | 1-factor 12-item baseline model | 561.45 | 54 | .81 | .13 |
| AAG-P2 | 3-factor 12-item model. | a | a | a | a |
| AAG-P3 | 2-factor 11-item model | 342.78 | 43 | .87 | .11 |
| AAG-P4 | 2-factor 9-item model | 187.99 | 26 | .90 | .11 |
| AAG-P5 | 3-factor 9-item model | a | a | a | a |
| AAG-P3R | 2-factor 7-item model | 39.87 | 13 | 0.06 | 0.98 |
| AAG-P1R | 1-factor 7-item model | 153.49 | 14 | 0.14 | 0.90 |
| Analyses of cross-validation sample | | | | | |
| AAG-F3R | 2-factor 8-item model | 64.18 | 19 | 0.04 | 0.98 |
| AAG-F1R | 1-factor 8-item model | 129.23 | 20 | 0.06 | 0.95 |

*Note*. Pilot sample (*N* = 549). Cross-validation sample (*N* = 1,674). *df* = degrees of freedom. RMSEA = root mean square error of approximation. CFI = comparative fit index.

^a^Model fit is not reported for models that resulted in improper estimates (e.g., estimated correlations greater than 1.00).

**Table C-2**

*Standardized Loadings and Standard Errors (SE) for One-Factor Baseline Model of the General Attitudes Toward Firearms Scale (Model AAG-P1)*

| Item | Label | Loading | *SE* |
| --- | --- | --- | --- |
| AAG01 | Firearms are necessary. | 0.83 | 0.019 |
| AAG03 | Firearms are the best way to protect yourself. | 0.80 | 0.021 |
| AAG07 | Firearms do more harm than good. | -0.78 | 0.032 |
| AAG05 | Firearms don't kill people. People kill people. | 0.74 | 0.026 |
| AAG02 | I have the right to own a firearm. | 0.71 | 0.025 |
| AAG06 | Firearms are not good or bad. | 0.68 | 0.033 |
| AAG09 | Firearms are scary. | -0.61 | 0.037 |
| AAG11 | No one should own a firearm. | -0.66 | 0.036 |
| AAG04 | Firearms are tools. | 0.60 | 0.033 |
| AAG10 | Everyone should own a firearm. | 0.58 | 0.036 |
| AAG12 | Everyone should be able to own a firearm. | 0.51 | 0.037 |
| AAG08 | Firearms are weapons. | -0.24 | 0.048 |

*Note.* Analyses based on pilot sample (*N* = 549). Items are listed in order of decreasing absolute value of loadings within each factor.

**Table C-3**

*Standardized Loadings and Standard Errors (SE) for a Hypothesized Three-factor Model of the General Attitudes Toward Firearms Scale (Model AAG-P2)*

| Item | Label | Loading | *SE* |
| --- | --- | --- | --- |
| Right to Own |  |  |  |
| AAG02 | I have the right to own a firearm. | 0.69 | 0.030 |
| AAG11 | No one should own a firearm. | -0.64 | 0.036 |
| AAG10 | Everyone should own a firearm. | 0.57 | 0.034 |
| AAG12 | Everyone should be able to own a firearm. | 0.48 | 0.039 |
| Functionality |  |  |  |
| AAG01 | Firearms are necessary. | 0.85 | 0.018 |
| AAG03 | Firearms are the best way to protect yourself. | 0.81 | 0.020 |
| AAG04 | Firearms are tools. | 0.60 | 0.034 |
| AAG05 | Firearms don't kill people. People kill people. | 0.74 | 0.027 |
| AAG06 | Firearms are not good or bad. | 0.68 | 0.033 |
| Fear & Harm |  |  |  |
| AAG07 | Firearms do more harm than good. | 0.95 | 0.023 |
| AAG09 | Firearms are scary. | 0.71 | 0.028 |
| AAG08 | Firearms are weapons. | 0.37 | 0.038 |

*Note.* Analyses based on pilot sample (*N* = 549). Items are listed in order of decreasing absolute value of loadings within each factor. Model resulted in an improper solution (e.g., the estimated correlation between two factors was greater than 1.00).

**Table C-4**

*Standardized Loadings and Standard Errors (SE) for a Hypothesized Two-factor 11-item Model of the General Attitudes Toward Firearms Scale (Model AAG-P3)*

| Item | Label | Loading | *SE* |
| --- | --- | --- | --- |
| Positive Attitudes Toward Firearms | | | |
| AAG01 | Firearms are necessary. | 0.85 | 0.018 |
| AAG02 | I have the right to own a firearm. | 0.71 | 0.025 |
| AAG03 | Firearms are the best way to protect yourself. | 0.84 | 0.019 |
| AAG04 | Firearms are tools. | 0.59 | 0.035 |
| AAG05 | Firearms don't kill people. People kill people. | 0.72 | 0.028 |
| AAG12 | Everyone should be able to own a firearm. | 0.52 | 0.037 |
| AAG10 | Everyone should own a firearm. | 0.60 | 0.036 |
| Negative Attitudes Toward Firearms | | | |
| AAG07 | Firearms do more harm than good. | 0.92 | 0.020 |
| AAG08 | Firearms are weapons. | 0.36 | 0.04 |
| AAG09 | Firearms are scary. | 0.73 | 0.025 |
| AAG11 | No one should own a firearm. | 0.69 | 0.030 |
| AAG06 | Firearms are not good or bad. | 0.68 | 0.033 |

*Note.* Analyses based on pilot sample (*N* = 549). Items are listed in order of decreasing absolute value of loadings within each factor. Item AAG06 was not included in the model.

**Table C-5**

*Standardized Loadings and Standard Errors (SE) for a Hypothesized Two-factor 9-item Model of the General Attitudes Toward Firearms Scale (Model AAG-P4)*

| Item | Label | Loading | *SE* |
| --- | --- | --- | --- |
| Firearms are Useful | | | |
| AAG02 | I have the right to own a firearm. | 0.73 | 0.027 |
| AAG03 | Firearms are the best way to protect yourself. | 0.77 | 0.024 |
| AAG06 | Firearms are not good or bad. | 0.69 | 0.036 |
| AAG12 | Everyone should be able to own a firearm. | 0.50 | 0.038 |
| AAG04 | Firearms are tools. | 0.64 | 0.030 |
| Firearms are a Problem | | | |
| AAG05 | Firearms don't kill people. People kill people. | 0.70 | 0.04 |
| AAG07 | Firearms do more harm than good. | -0.85 | 0.02 |
| AAG09 | Firearms are scary. | -0.70 | 0.03 |
| AAG11 | No one should own a firearm. | -0.71 | 0.03 |

*Note.* Analyses based on pilot sample (*N* = 549). Items are listed in order of decreasing absolute value of loadings within each factor. Correlation between the two factors was .89.

**Table C-6**

*Standardized Loadings and Standard Errors (SE) for the Revised Two-factor Model of the General Attitudes Toward Firearms Scale (Model AAG-P3R and AAGF3R)*

|  |  | Pilot sample  (*N* = 549) | | Cross-validation sample  (*N* = 1,674) | |
| --- | --- | --- | --- | --- | --- |
| Item | Label | Loading | *SE* | Loading | *SE* |
| Positive Attitudes Toward Firearms | |  |  |  |  |
| AAG01 | Firearms are necessary. | 0.86 | 0.018 | 0.84 | 0.014 |
| AAG02 | I have the right to own a firearm. | 0.73 | 0.024 | 0.69 | 0.035 |
| AAG03 | Firearms are the best way to protect yourself. | 0.82 | 0.020 | 0.80 | 0.015 |
| AAG04 | Firearms are tools. | 0.60 | 0.035 | 0.58 | 0.028 |
| AAG05 | Firearms don't kill people. People kill people. | 0.72 | 0.030 | 0.74 | 0.017 |
| Negative Attitudes Toward Firearms | |  |  |  |  |
| AAG07 | Firearms do more harm than good. | 0.96 | 0.026 | 0.85 | 0.019 |
| AAG09 | Firearms are scary. | 0.70 | 0.029 | 0.74 | 0.019 |
| AAG13 | Firearms are bad. | a | a | 0.86 | 0.013 |

*Note.* Correlation between the two factors was -.76 in the pilot sample, and -.90 in the cross-validation sample.

^a^Item not included in the cross-validation sample.

**Table C-7**

*Standardized Loadings and Standard Errors (SE) for a Hypothesized One-factor Model of the General Attitudes Toward Firearms Scale (Model AAG-P1R and AAGF1R)*

|  |  | Pilot sample  (*N* = 549) | | Cross-validation sample  (*N* = 1,674) | |
| --- | --- | --- | --- | --- | --- |
| Item | Label | Loading | *SE* | Loading | *SE* |
| AAG01 | Firearms are necessary. | 0.84 | 0.020 | 0.81 | 0.015 |
| AAG02 | I have the right to own a firearm. | 0.72 | 0.024 | 0.67 | 0.032 |
| AAG03 | Firearms are the best way to protect yourself. | 0.82 | 0.021 | 0.78 | 0.016 |
| AAG04 | Firearms are tools. | 0.61 | 0.034 | 0.58 | 0.027 |
| AAG05 | Firearms don't kill people. People kill people. | 0.72 | 0.028 | 0.73 | 0.017 |
| AAG07 | Firearms do more harm than good. | -0.75 | 0.036 | -0.83 | 0.018 |
| AAG09 | Firearms are scary. | -0.58 | 0.042 | -0.70 | 0.021 |
| AAG13 | Firearms are bad. | a | a | -0.83 | 0.014 |

^a^Item not included in the cross-validation sample.

# **Appendix D**

## Evaluation of Beliefs About Defensive Firearm Use Measure

***Models Tested***

Confirmatory Factor Analyses (CFA) of data from the pilot sample evaluated a 1-factor baseline model (BLG-P1) and three hypothesized models proposed by the research team. These included: a 2-factor model with factors reflecting General Beliefs and Beliefs About Use in Specific Situations; a 3-factor model using 21 items with factors representing Imminent Defensive Escalation, Respect Enforcement, and Conditions of Use; and a 4-factor model using 21 items with factors representing Defensive Escalation, Conditions of Use, Shooting/Killing, and Respect Enforcement. Fit indices for each of these models are reported in Table D-1 and the standardized loadings for each of these models are reported in tables D-2 to D-5. The 1-factor model and the proposed 2-factor, 3-factor, and 4-factor models did not adequately fit the data (see Table D-1).

Given the poor fit of the one-factor and three hypothesized models, exploratory factor analyses (EFA) using a geomin oblique rotation were used to identify potential structures that best represented the structure of the measure. Although the EFA identified five factors associated with eigenvalues greater than 1.00 (5.76, 3.84, 2.21, 1.38, 1.15), values leveled off after four factors. The 3-factor EFA model identified four items with poor loadings on all three factors. The remaining items had good specificity such that each of the three factors was represented by 3 to 6 items with fair to excellent loadings. The 4-factor EFA model identified only two items with poor loadings on all four factors. The remaining items had good specificity such that each of the four factors was represented by 3 to 8 items with fair to excellent loadings. CFAs were next conducted based on the 3-factor and 4-factor structures identified by the EFA. Within the CFA models each item was allowed to load on only one of the factors based on patterns identified by the EFA (loadings on all other factors constrained to zero). These analyses excluded items with poor loadings on every factor in the EFA. The resulting 3-factor 18-item model (BLG-P5) did not adequately fit the data. The 4-factor 20-item model (BLG-P6) had an acceptable fit based on the RMSEA, but a poor fit based on the CFI. All of the items had good specificity, with fair to excellent loadings (see Table D-7).

***Final Model***

Selection of items to include in the cross-validation sample was based on a review of the results of the 4-factor model suggested by the exploratory analysis of the pilot sample. Items were selected based on factor loadings and substantive decisions. None of the items from the Conditions of Use factor were included due to the team’s consensus that this factor assessed rules of responsible use taught in basic firearm safety courses, rather than beliefs specific to using firearms against other people. A CFA indicated that the resulting 3-factor 10-item model had an acceptable fit in both the pilot and cross-validation samples (see Model BLG-P6R inTable D-1). All standardized loadings were fair to excellent for all three factors in both samples, with one exception (see Table D-8). Within this model, the Compensatory Force factor was moderately to highly correlated with the Respect Enforcement and Defensive Escalation factors (*r*s = .45 and .68, respectively). The Respect Enforcement and Defensive Escalation factors had a significant, but low correlation (*r* = .10). Reliability coefficients (omega) for the Respect Enforcement, Defensive Escalation, and Compensatory Force factors were .82, .81, and .66, respectively. **Table D-1**

*Fit Indices for Competing Models of the structure of the Attitudes Toward Defensive Firearm Use Measure*

| Model | Description | χ2 | *df* | RMSEA | CFI |
| --- | --- | --- | --- | --- | --- |
| Analyses of pilot sample | | | | | |
| BLG-P1 | 1-factor model | 2525.70 | 209 | 0.142 | 0.463 |
| BLG-P2 | 2-factor hypothesized model | 2438.20 | 208 | 0.140 | 0.483 |
| BLG-P3 | 3-factor 21-item hypothesized model | 1447.6 | 186 | 0.111 | 0.684 |
| BLG-P4 | 4-factor 21-item hypothesized model | 1697.70 | 183 | 0.123 | 0.621 |
| BLG-P5 | 3-factor 18-item model based on 3-factor EFA | 1033.67 | 132 | 0.112 | 0.750 |
| BLG-P6 | 4-factor 20-item model based on 4-factor EFA | 856.88 | 164 | 0.088 | 0.823 |
| PLG-P6R | 3-factor 10-item model based on BLG-P6 | 166.01 | 32 | 0.088 | 0.904 |
| Analyses of cross-validation sample | |  |  |  |  |
| PLG-F6R | 3-factor 10-item model | 254.07 | 32 | 0.065 | 0.918 |

*Note.* Pilot sample (*N* = 549). Cross-validation sample (*N* = 1,674). df =degrees of freedom. RMSEA=root mean square error of approximation. CFI = comparative fit index.

**Table D-2**

*Standardized Loadings and Standard Errors (SE) for One-Factor Baseline Model of the Beliefs Toward Defensive Firearm Use Measure (Model BLG-P1)*

| Item | Label | Loading | *SE* |
| --- | --- | --- | --- |
| BLG01 | You should only pull your firearm if you intend to use it. | 0.10 | 0.05 |
| BLG02 | You have to accept the possibility that you might shoot someone if you carry a firearm. | 0.33 | 0.05 |
| BLG03 | Pulling a firearm should be a last resort in a conflict. | 0.48 | 0.06 |
| BLG04 | You should warn someone before you pull a firearm on them. | 0.17 | 0.04 |
| BLG05 | If you shoot your firearm at someone, you should shoot to kill. | -0.21 | 0.04 |
| BLG06 | You should only pull a firearm if someone's life is in danger. | 0.25 | 0.05 |
| BLG07 | If you shoot your firearm at someone, you should try to stop them without killing them. | 0.20 | 0.04 |
| BLG08 | Showing that you have a firearm can make everyone back down. | -0.22 | 0.04 |
| BLG09 | People will not mess with you if they know you have a firearm. | -0.30 | 0.04 |
| BLG10 | If someone disrespects a female family member, then it is okay to shoot at them. | -0.87 | 0.02 |
| BLG11 | If someone verbally threatens your family, it's okay to shoot at them. | -0.85 | 0.03 |
| BLG12 | If someone embarrasses you, then it is okay to tell them you have a firearm. | -0.78 | 0.03 |
| BLG13 | If you find out your partner is cheating on you, it's okay to go get a firearm. | -0.88 | 0.03 |
| BLG14 | If your partner tries to leave you, it's okay to point a firearm at them. | -0.89 | 0.02 |
| BLG15 | If someone makes you feel threatened, it's okay to tell them you have a firearm. | -0.38 | 0.04 |
| BLG16 | If someone flashed a firearm at you, it's okay to go get your firearm and bring it back. | -0.49 | 0.04 |
| BLG17 | If someone bigger is coming at you, it's okay to tell them you have a firearm. | -0.27 | 0.04 |
| BLG18 | If someone breaks into your house, it's okay to point a firearm at them. | 0.13 | 0.06 |
| BLG19 | If someone killed a person who you're close to, it's okay to go kill them. | -0.61 | 0.04 |
| BLG20 | If you see someone getting attacked, it's okay to shoot the attacker. | -0.19 | 0.05 |
| BLG21 | If someone points a firearm at you, it's okay to shoot them. | -0.06 | 0.05 |
| BLG22 | If someone breaks into your house, it's okay to go get a firearm. | 0.13 | 0.06 |

*Note.* Analyses based on pilot sample (*N* = 547). Items are listed in order of decreasing absolute value of loadings within each factor.

**Table D-3**

*Standardized Loadings and Standard Errors (SE) for a Hypothesized Two-factor Model of the Beliefs Toward Defensive Firearm Use Scale (Model BLG-P2)*

| Item | Label | Loading | *SE* |
| --- | --- | --- | --- |
| General Beliefs | |  |  |
| BLG03 | Pulling a firearm should be a last resort in a conflict. | 0.80 | 0.055 |
| BLG06 | You should only pull a firearm if someone's life is in danger. | 0.54 | 0.060 |
| BLG02 | You have to accept the possibility that you might shoot someone if you carry a firearm. | 0.46 | 0.056 |
| BLG07 | If you shoot your firearm at someone, you should try to stop them without killing them. | 0.41 | 0.076 |
| BLG04 | You should warn someone before you pull a firearm on them. | 0.37 | 0.066 |
| BLG01 | You should only pull your firearm if you intend to use it. | 0.32 | 0.070 |
| BLG05 | If you shoot your firearm at someone, you should shoot to kill. | -0.22 | 0.089 |
| BLG09 | People will not mess with you if they know you have a firearm. | -0.15 | 0.081 |
| BLG08 | Showing that you have a firearm can make everyone back down. | -0.08 | 0.075 |
| Situation Specific Beliefs | |  |  |
| BLG03 | Pulling a firearm should be a last resort in a conflict. | 0.80 | 0.055 |
| BLG06 | You should only pull a firearm if someone's life is in danger. | 0.54 | 0.060 |
| BLG02 | You have to accept the possibility that you might shoot someone if you carry a firearm. | 0.46 | 0.056 |
| BLG07 | If you shoot your firearm at someone, you should try to stop them without killing them. | 0.41 | 0.076 |
| BLG04 | You should warn someone before you pull a firearm on them. | 0.37 | 0.066 |
| BLG01 | You should only pull your firearm if you intend to use it. | 0.32 | 0.070 |
| BLG05 | If you shoot your firearm at someone, you should shoot to kill. | -0.22 | 0.089 |
| BLG09 | People will not mess with you if they know you have a firearm. | -0.15 | 0.081 |
| BLG08 | Showing that you have a firearm can make everyone back down. | -0.08 | 0.075 |
| BLG03 | Pulling a firearm should be a last resort in a conflict. | 0.80 | 0.055 |
| BLG06 | You should only pull a firearm if someone's life is in danger. | 0.54 | 0.060 |
| BLG02 | You have to accept the possibility that you might shoot someone if you carry a firearm. | 0.46 | 0.056 |
| BLG07 | If you shoot your firearm at someone, you should try to stop them without killing them. | 0.41 | 0.076 |

*Note.* Analyses based on pilot sample (*N* = 547). Items are listed in order of decreasing absolute value of loadings within each factor. The correlation between the two factors is -.56.

**Table D-4**

*Standardized Loadings and Standard Errors (SE) for a Hypothesized Three-factor 21-item Model of the Beliefs Toward Defensive Firearm Use Scale (Model BLG-P3)*

| Item | Label | Loading | *SE* |
| --- | --- | --- | --- |
| Defensive Escalation | |  |  |
| BLG18 | If someone breaks into your house, it's okay to point a firearm at them. | 0.87 | 0.027 |
| BLG22 | If someone breaks into your house, it's okay to go get a firearm. | 0.87 | 0.022 |
| BLG21 | If someone points a firearm at you, it's okay to shoot them. | 0.65 | 0.034 |
| BLG20 | If you see someone getting attacked, it's okay to shoot the attacker. | 0.55 | 0.038 |
| BLG06 | You should only pull a firearm if someone's life is in danger. | 0.43 | 0.056 |
| BLG17 | If someone bigger is coming at you, it's okay to tell them you have a firearm. | 0.34 | 0.049 |
| BLG15 | If someone makes you feel threatened, it's okay to tell them you have a firearm. | 0.26 | 0.048 |
| Respect Enforcement | |  |  |
| BLG14 | If your partner tries to leave you, it's okay to point a firearm at them. | 0.89 | 0.025 |
| BLG13 | If you find out your partner is cheating on you, it's okay to go get a firearm. | 0.88 | 0.035 |
| BLG10 | If someone disrespects a female family member, then it is okay to shoot at them. | 0.88 | 0.023 |
| BLG11 | If someone verbally threatens your family, it's okay to shoot at them. | 0.86 | 0.026 |
| BLG12 | If someone embarrasses you, then it is okay to tell them you have a firearm. | 0.78 | 0.035 |
| BLG19 | If someone killed a person who you're close to, it's okay to go kill them. | 0.60 | 0.044 |
| BLG16 | If someone flashed a firearm at you, it's okay to go get your firearm and bring it back. | 0.47 | 0.037 |
| BLG09 | People will not mess with you if they know you have a firearm. | 0.28 | 0.040 |
| Conditions of Use | |  |  |
| BLG03 | Pulling a firearm should be a last resort in a conflict. | 0.83 | 0.054 |
| BLG02 | You have to accept the possibility that you might shoot someone if you carry a firearm. | 0.47 | 0.064 |
| BLG01 | You should only pull your firearm if you intend to use it. | 0.37 | 0.056 |
| BLG07 | If you shoot your firearm at someone, you should try to stop them without killing them. | 0.31 | 0.057 |
| BLG04 | You should warn someone before you pull a firearm on them. | 0.30 | 0.058 |
| BLG08 | Showing that you have a firearm can make everyone back down. | -0.04 | 0.059 |

*Note.* Analyses based on pilot sample (*N* = 547). Items are listed in order of decreasing absolute value of loadings within each factor. The Respect Enforcement factor was negatively correlated with the Defensive Escalation (r= -.08, *p* = .176) and Conditions of Use factors (*r* = -.56, *p* < .001). The Defensive Escalation and Conditions of Use factors were positively correlated (r = .41, *p* < .001).

**Table D-5**

*Standardized Loadings and Standard Errors (SE) for a Hypothesized Four-factor 21-item Model of the Beliefs About Defensive Firearm Use Scale (Model BLG-P4)*

| Item | Label | Loading | *SE* |
| --- | --- | --- | --- |
| Defensive Escalation | |  |  |
| BLG18 | If someone breaks into your house, it's okay to point a firearm at them. | 0.88 | 0.029 |
| BLG22 | If someone breaks into your house, it's okay to go get a firearm. | 0.87 | 0.026 |
| BLG17 | If someone bigger is coming at you, it's okay to tell them you have a firearm. | 0.33 | 0.051 |
| BLG15 | If someone makes you feel threatened, it's okay to tell them you have a firearm. | 0.25 | 0.050 |
| BLG16 | If someone flashed a firearm at you, it's okay to go get your firearm and bring it back. | 0.19 | 0.052 |
| Conditions of Use | |  |  |
| BLG03 | Pulling a firearm should be a last resort in a conflict. | 0.81 | 0.045 |
| BLG06 | You should only pull a firearm if someone's life is in danger. | 0.59 | 0.051 |
| BLG02 | You have to accept the possibility that you might shoot someone if you carry a firearm. | 0.45 | 0.059 |
| BLG01 | You should only pull your firearm if you intend to use it. | 0.41 | 0.062 |
| BLG04 | You should warn someone before you pull a firearm on them. | 0.25 | 0.063 |
| BLG05 | If you shoot your firearm at someone, you should shoot to kill. | -0.03 | 0.078 |
| Shooting/Killing | |  |  |
| BLG10 | If someone disrespects a female family member, then it is okay to shoot at them. | 0.92 | 0.016 |
| BLG11 | If someone verbally threatens your family, it's okay to shoot at them. | 0.91 | 0.02 |
| BLG19 | If someone killed a person who you're close to, it's okay to go kill them. | 0.61 | 0.045 |
| BLG20 | If you see someone getting attacked, it's okay to shoot the attacker. | 0.22 | 0.047 |
| BLG21 | If someone points a firearm at you, it's okay to shoot them. | 0.10 | 0.045 |
| Respect Enforcement | |  |  |
| BLG14 | If your partner tries to leave you, it's okay to point a firearm at them. | 0.92 | 0.019 |
| BLG13 | If you find out your partner is cheating on you, it's okay to go get a firearm. | 0.90 | 0.030 |
| BLG12 | If someone embarrasses you, then it is okay to tell them you have a firearm. | 0.77 | 0.037 |
| BLG09 | People will not mess with you if they know you have a firearm. | 0.28 | 0.039 |
| BLG08 | Showing that you have a firearm can make everyone back down. | 0.19 | 0.036 |

*Note.* Analyses based on pilot sample (*N* = 547). Items are listed in order of decreasing absolute value of loadings within each factor. All correlations among the factors were significant except for the correlation between Shooting/Killing, and Defensive Escalation. Conditions of Use was negatively correlated with Shooting/Killing (*r* = -.45) and Respect Enforcement (*r* = -.57) and positively correlated with Defensive Escalation (*r* = .48). Defensive Escalation and the Respect Enforcement factors were negatively correlated (*r* = -.15). Shooting/Killing factor was positively correlated with Respect Enforcement (*r* = .89). Shooting was negative correlated with Defensive Escalation and Conditions of Use.

**Table D-6**

*Standardized Loadings and Standard Errors (SE) for a Hypothesized Three-factor 18-item Model of the Beliefs Toward Defensive Firearm Use Scale (Model BLG-P5)*

| Item | Label | Loading | *SE* |
| --- | --- | --- | --- |
| Respect & Retaliation | |  |  |
| BLG14 | If your partner tries to leave you, it's okay to point a firearm at them. | 0.88 | 0.027 |
| BLG10 | If someone disrespects a female family member, then it is okay to shoot at them. | 0.88 | 0.022 |
| BLG13 | If you find out your partner is cheating on you, it's okay to go get a firearm. | 0.87 | 0.035 |
| BLG11 | If someone verbally threatens your family, it's okay to shoot at them. | 0.86 | 0.025 |
| BLG12 | If someone embarrasses you, then it is okay to tell them you have a firearm. | 0.78 | 0.035 |
| BLG19 | If someone killed a person who you're close to, it's okay to go kill them. | 0.60 | 0.043 |
| BLG16 | If someone flashed a firearm at you, it's okay to go get your firearm and bring it back. | 0.48 | 0.038 |
| BLG15 | If someone makes you feel threatened, it's okay to tell them you have a firearm. | 0.37 | 0.034 |
| Rules of Use | |  |  |
| BLG07 | If you shoot your firearm at someone, you should try to stop them without killing them. | 0.83 | 0.057 |
| BLG05 | If you shoot your firearm at someone, you should shoot to kill. | -0.74 | 0.063 |
| BLG04 | You should warn someone before you pull a firearm on them. | 0.54 | 0.047 |
| Defense of Self and Others | |  |  |
| BLG22 | If someone breaks into your house, it's okay to go get a firearm. | 0.88 | 0.020 |
| BLG18 | If someone breaks into your house, it's okay to point a firearm at them. | 0.87 | 0.025 |
| BLG21 | If someone points a firearm at you, it's okay to shoot them. | 0.64 | 0.034 |
| BLG20 | If you see someone getting attacked, it's okay to shoot the attacker. | 0.53 | 0.038 |
| BLG06 | You should only pull a firearm if someone's life is in danger. | 0.43 | 0.056 |
| BLG03 | Pulling a firearm should be a last resort in a conflict. | 0.37 | 0.060 |
| BLG17 | If someone bigger is coming at you, it's okay to tell them you have a firearm. | 0.31 | 0.047 |

*Note.* Analyses based on pilot sample (*N* = 547) based on pattern identified by an exploratory factor analysis. Items are listed in order of decreasing absolute value of loadings within each factor. This model did not include four items from the original measure that did not have a clear pattern of loadings in the three-factor exploratory model (i.e., BLG01, BLG02, BLG08, BLG09). The correlation between Factor 1 and Factor 2 and Factor 3 were -.24 and -.12, respectively. The correlation between Factor 2 and Factor 3 was -.18.

**Table D-7**

*Standardized Loadings and Standard Errors (SE) for a Four-factor 20-item Model of the Beliefs About Defensive Firearm Use Scale (Model BLG-P6)*

| Item | Label | Loading | *SE* |
| --- | --- | --- | --- |
| Respect Enforcement | |  |  |
| BLG14 | If your partner tries to leave you, it's okay to point a firearm at them. | 0.89 | 0.03 |
| BLG13 | If you find out your partner is cheating on you, it's okay to go get a firearm. | 0.88 | 0.04 |
| BLG10 | If someone disrespects a female family member, then it is okay to shoot at them. | 0.87 | 0.02 |
| BLG11^a^ | If someone verbally threatens your family, it's okay to shoot at them. | 0.86 | 0.03 |
| BLG12^a^ | If someone embarrasses you, then it is okay to tell them you have a firearm. | 0.78 | 0.04 |
| BLG19 | If someone killed a person who you're close to, it's okay to go kill them. | 0.60 | 0.04 |
| Conditions of Use | |  |  |
| BLG07^a^ | If you shoot your firearm at someone, you should try to stop them without killing them. | 0.83 | 0.05 |
| BLG05^a^ | If you shoot your firearm at someone, you should shoot to kill. | -0.74 | 0.06 |
| BLG04^a^ | You should warn someone before you pull a firearm on them. | 0.54 | 0.05 |
| Defensive Escalation | |  |  |
| BLG22 | If someone breaks into your house, it's okay to go get a firearm. | 0.88 | 0.02 |
| BLG18^a^ | If someone breaks into your house, it's okay to point a firearm at them. | 0.86 | 0.03 |
| BLG21 | If someone points a firearm at you, it's okay to shoot them. | 0.65 | 0.03 |
| BLG20 | If you see someone getting attacked, it's okay to shoot the attacker. | 0.55 | 0.04 |
| BLG06^a^ | You should only pull a firearm if someone's life is in danger. | 0.42 | 0.06 |
| BLG01^a^ | You should only pull your firearm if you intend to use it. | 0.39 | 0.05 |
| Compensatory Force | |  |  |
| BLG15 | If someone makes you feel threatened, it's okay to tell them you have a firearm. | 0.73 | 0.03 |
| BLG16 | If someone flashed a firearm at you, it's okay to go get your firearm and bring it back. | 0.71 | 0.03 |
| BLG17^a^ | If someone bigger is coming at you, it's okay to tell them you have a firearm. | 0.68 | 0.04 |
| BLG09 | People will not mess with you if they know you have a firearm. | 0.50 | 0.05 |
| BLG08^a^ | Showing that you have a firearm can make everyone back down. | 0.48 | 0.06 |

*Note.* Analyses based on pilot sample (*N* = 547) based on pattern identified by an exploratory factor analysis. Items are listed in order of decreasing absolute value of loadings within each factor. This model did not include two items from the original measure that did not have a clear pattern of loadings in the 4-factor exploratory model (i.e., BLG02, BLG03). The correlations between the Compensatory Force and Respect Enforcement, Conditions of Use and Defensive Escalation factors were .50, -.06, and .34, respectively. Correlations between the Defensive Escalation and the Respect Enforcement and Conditions of Use factors were -.12 and -.21, respectively. The correlation between the Conditions of Use and Respect factors was -.24.

^a^Identifies items that were excluded from the final 10-item model (Model BLG-5R).

**Table D-8**

*Standardized Loadings and Standard Errors for the Final 3-Factor 10-item CFA Model (BLG-6R) Beliefs About Defensive Firearm Use Measure*

|  |  | Pilot sample  (*N* = 549) | | Cross-validation sample  (*N* = 1,674) | |
| --- | --- | --- | --- | --- | --- |
| Item | Label | Loading | *SE* | Loading | *SE* |
| Respect Enforcement | |  |  |  |  |
| BLG10 | If someone disrespects a female family member, then it is okay to shoot at them. | 0.81 | 0.03 | 0.77 | 0.03 |
| BLG13 | If you find out your partner is cheating on you, it's okay to go get a firearm. | 0.91 | 0.03 | 0.88 | 0.02 |
| BLG14 | If your partner tries to leave you, it's okay to point a firearm at them. | 0.92 | 0.02 | 0.84 | 0.02 |
| BLG19 | If someone killed a person who you're close to, it's okay to go kill them. | 0.58 | 0.05 | 0.56 | 0.03 |
| Defensive Escalation | |  |  |  |  |
| BLG20 | If you see someone getting attacked, it's okay to shoot the attacker. | 0.66 | 0.04 | 0.72 | 0.02 |
| BLG21 | If someone points a firearm at you, it's okay to shoot them. | 0.78 | 0.04 | 0.83 | 0.02 |
| BLG22 | If someone breaks into your house, it's okay to go get a firearm. | 0.71 | 0.04 | 0.74 | 0.02 |
| Compensatory Force | |  |  |  |  |
| BLG15 | If someone makes you feel threatened, it's okay to tell them you have a firearm. | 0.69 | 0.04 | 0.74 | 0.02 |
| BLG16 | If someone flashed a firearm at you, it's okay to go get your firearm and bring it back. | 0.78 | 0.04 | 0.71 | 0.02 |
| BLG09 | People will not mess with you if they know you have a firearm. | 0.41 | 0.05 | 0.38 | 0.04 |

*Note.* Correlations between Respect and Defense and Compensatory Force factors were .10 and .45, respectively. The correlation between Defensive Escalation and Compensatory Force factors was .68.

# **Appendix E**

## Evaluation of Prescriptive Norms for Threat Response Measure

***Models Tested***

Confirmatory Factor Analyses (CFA) of data from the pilot sample evaluated a baseline 1-factor model (AAP-P1) and three models hypothesized by the research team. Hypothesized models included two different 3-factor models, and a 2-factor model. Fit indices for each of these models are reported in Table E-1 and the standardized loadings for each of these models are reported in tables E-2 to E-5). The 1-factor model did not adequately fit the data. Both of the 3-factor models (CAS-P2 and CAS-P3) fit the data poorly and resulted in improper solutions (i.e., the latent variable covariance matrix was not positive definite). The 2-factor hypothesized model (CAS-P4), which specified factors representing Prescriptive Norms for Defense and Protection, and Prescriptive Norms for Reputation and Respect, appeared the most promising. Review of the standardized loadings indicated only 1 loading that was less than .45, and all but one of the remaining loadings were good to excellent (see Table E-6).

***Final Model***

After reviewing the loadings and content of each factor an alternative version of the 2-factor model was evaluated (Model CAS-P4R) in which three items were removed (i.e., CAS03, CAS05, CAS09). This resulted in a better fitting model in the pilot sample that also had an acceptable fit in the cross-validation sample (see Table E-1). Standardized loadings for this model were all good to excellent (see Table E-6). The two factors were moderately correlated in the pilot and cross-validation samples (*r* = .49 and .41, respectively). Reliability coefficients (omega) in the pilot and cross-validation samples were .84 in both samples for the Prescriptive Norms for Defense and Protection, and were .84 and .81, respectively for the Prescriptive Norms for Reputation and Respect factors.

**Table E-1**

*Fit Indices for Competing Models of the structure of the Attitudes Toward Firearm-Related Policies Measure*

| Model | Description | χ2 | *df* | RMSEA | CFI |
| --- | --- | --- | --- | --- | --- |
| Analyses of pilot sample | | | | | |
| CAS-P1 | 1-factor 9-item model | 430.82 | 27 | 0.165 | 0.674 |
| CAS-P2 | 3-factor 9-item model | 317.43 | 24 | 0.149 | 0.763 |
| CAS-P3 | 3-factor 9-item model | 281.74 | 24 | 0.140 | 0.792 |
| CAS -P4 | 2-factor 9-item model | 155.32 | 26 | 0.095 | 0.896 |
| CAS -P4R | 2-factor 6-item model | 42.88 | 8 | 0.089 | 0.953 |
| Analyses of cross-validation sample | |  |  |  |  |
| CAS -F4R | 2-factor 6-item model | 118.36 | 8 | 0.091 | 0.931 |

*Note.* Pilot sample (*N* = 549). Cross-validation sample (*N* = 1,674). *df* =degrees of freedom. RMSEA = root mean square error of approximation. CFI = comparative fit index.

**Table E-2**

*Standardized Loadings and Standard Errors (SE) for One-Factor Baseline Model of the Cultural Norms About Protection and Defense Scale (Model CAS-P1)*

| Item | Label | Loading | *SE* |
| --- | --- | --- | --- |
| CAS01 | People should be ready to defend themselves. | 0.81 | 0.046 |
| CAS02 | People should be prepared to defend their homes. | 0.85 | 0.030 |
| CAS03 | People should stand up for themselves. | 0.78 | 0.033 |
| CAS04 | Men should protect the women in their lives. | 0.66 | 0.041 |
| CAS05 | People should be loyal to their families. | 0.56 | 0.052 |
| CAS06 | People need to keep up a reputation to stop others from messing with them. | 0.45 | 0.051 |
| CAS07 | People should always stand their ground. | 0.56 | 0.045 |
| CAS08 | When people get disrespected, they need to do something about it. | 0.38 | 0.057 |
| CAS09 | People should get the respect that they deserve. | 0.29 | 0.053 |

*Note.* Analyses based on pilot sample (*N* = 549). Items are listed in order of decreasing absolute value of loadings.

**Table E-3**

*Standardized Loadings and Standard Errors (SE) for a Hypothesized Three-factor Model (Model CAS-P2) of the Cultural Norms for Protection and Defense Scale*

| Item | Label | Loading | *SE* |
| --- | --- | --- | --- |
| Self Defense | |  |  |
| CAS01 | People should be ready to defend themselves. | 0.81 | 0.049 |
| CAS03 | People should stand up for themselves. | 0.77 | 0.038 |
| CAS07 | People should always stand their ground. | 0.55 | 0.047 |
| Other Defense | |  |  |
| CAS02 | People should be prepared to defend their homes. | 0.87 | 0.022 |
| CAS04 | Men should protect the women in their lives. | 0.65 | 0.045 |
| CAS05 | People should be loyal to their families. | 0.55 | 0.056 |
| Respect |  |  |  |
| CAS06 | People need to keep up a reputation to stop others from messing with them. | 0.71 | 0.046 |
| CAS08 | When people get disrespected, they need to do something about it. | 0.81 | 0.038 |
| CAS09 | People should get the respect that they deserve. | 0.47 | 0.048 |

*Note.* Analyses based on pilot sample (*N* = 549). Items are listed in order of decreasing absolute value of loadings within each factor.

**Table E-4**

*Standardized Loadings and Standard Errors (SE) for a Hypothesized Three-factor Model (Model CAS-P3) of the of the Cultural Norms for Protection and Defense Scale*

| Item | Label | Loading | *SE* |
| --- | --- | --- | --- |
| Protect & Defend | |  |  |
| CAS01 | People should be ready to defend themselves. | 0.84 | 0.039 |
| CAS02 | People should be prepared to defend their homes. | 0.90 | 0.021 |
| CAS04 | Men should protect the women in their lives. | 0.62 | 0.048 |
| Respect | |  |  |
| CAS06 | People need to keep up a reputation to stop others from messing with them. | 0.69 | 0.043 |
| CAS08 | When people get disrespected, they need to do something about it. | 0.81 | 0.031 |
| CAS09 | People should get the respect that they deserve. | 0.48 | 0.046 |
| Steadfastness | |  |  |
| CAS07 | People should always stand their ground. | 0.71 | 0.058 |
| CAS05 | People should be loyal to their families. | 0.51 | 0.066 |
| CAS03 | People should stand up for themselves. | 0.64 | 0.047 |

*Note.* Analyses based on pilot sample (*N* = 549). Items are listed in order of decreasing absolute value of loadings within each factor.

**Table E-5**

*Standardized Factor Loadings for Model 4 (2-factor 9-item model) of Cultural Norms for Protection and Defense Scale*

| Item | Label | Loading | *SE* |
| --- | --- | --- | --- |
| Prescriptive Norms for Defense and Protection | |  |  |
| CAS01 | People should be ready to defend themselves. | 0.83 | 0.042 |
| CAS02 | People should be prepared to defend their homes. | 0.88 | 0.023 |
| CAS03 | People should stand up for themselves. | 0.78 | 0.037 |
| CAS04 | Men should protect the women in their lives. | 0.64 | 0.045 |
| CAS05 | People should be loyal to their families. | 0.54 | 0.058 |
| Prescriptive Norms for Reputation and Respect | |  |  |
| CAS06 | People need to keep up a reputation to stop others from messing with them. | 0.73 | 0.034 |
| CAS07 | People should always stand their ground. | 0.81 | 0.027 |
| CAS08 | When people get disrespected, they need to do something about it. | 0.75 | 0.031 |
| CAS09 | People should get the respect that they deserve. | 0.42 | 0.047 |

*Note.* Analyses based on pilot sample (*N* = 549). Items are listed in order of decreasing absolute value of loadings within each factor. Correlation between factors was .51.

**Table E-6**

*Standardized Factor Loadings for Model 4R (2-factor 6-item model) of Cultural Norms for Protection and Defense in the Pilot and Cross-Validation Samples*

|  |  | Pilot sample  (*N* = 549) | | Cross-validation sample  (*N* = 1,674) | |
| --- | --- | --- | --- | --- | --- |
| Item | Label | Loading | *SE* | Loading | *SE* |
| Prescriptive Norms for Defense and Protection | |  |  |  |  |
| CAS01 | People should be ready to defend themselves. | 0.83 | 0.043 | 0.89 | 0.017 |
| CAS02 | People should be prepared to defend their homes. | 0.94 | 0.02 | 0.94 | 0.019 |
| CAS04 | Men should protect the women in their lives. | 0.58 | 0.044 | 0.55 | 0.034 |
| Prescriptive Norms for Reputation and Respect | |  |  |  |  |
| CAS06 | People need to keep up a reputation to stop others from messing with them. | 0.74 | 0.037 | 0.73 | 0.027 |
| CAS07 | People should always stand their ground. | 0.82 | 0.031 | 0.75 | 0.029 |
| CAS08 | When people get disrespected, they need to do something about it. | 0.73 | 0.031 | 0.73 | 0.026 |

*Note.* Correlations between the factors were .49 in the pilot sample, and .41 in the cross-validation sample.

# **Appendix F**

## Evaluation of Attitudes Toward Firearm-Related Policy Measure

***Models Tested***

Confirmatory factor analyses (CFA) of data from the pilot sample evaluated a baseline 1-factor model (AAP-P1) and a 4-factor model proposed by the research team (see tables 4 and 5). Fit indices for each of these models are reported in Table F-1 and the standardized loadings for each of these models are reported in tables F-2 and F-3. Neither model adequately fit the data and the 4-factor model resulted in an improper solution (i.e., correlation estimate exceeding 1.00). An exploratory factor analysis identified 2-factors with eigenvalues exceeding 1.00. The pattern of geomin-rotated loadings suggested separate factors representing Negative Attitudes Toward General Firearm Policies, and Positive Attitudes Toward Specific Firearm Policies (see Table F-4). A CFA based on these findings was conducted on a model (Model AAP-P3) that excluded four items that had poor loadings on both factors (see Table F-5). The resulting model had an adequate fit based on the RMSEA, but a CFI just below .90.

***Final Model***

The 2-factor model based on the EFA (Model AAP-P3) was refined by excluding an additional four items based primarily on substantive considerations. The resulting 12-item model (see Model AAP-P3R in Table F-6) adequately fit the data in the pilot sample and fit the data very well when it was cross-validated in the cross-validation sample (see Table F-1). Reliability coefficients (omega) in the pilot and cross-validation samples were .88 and .87, respectively for the Negative Attitudes Toward General Firearm Policies factor, and .91 in both samples for the Positive Attitudes Toward Specific Firearm Policies factor.

**Table F-1**

*Fit Indices for Competing Models of the structure of the Attitudes Toward Firearm-Related Policies Measure*

| Model | Description | χ2 | *df* | RMSEA | CFI |
| --- | --- | --- | --- | --- | --- |
| Analyses of pilot sample | | | | | |
| AAP-P1 | 1-factor 20-item model | 1286.96 | 170 | 0.110 | 0.749 |
| AAP-P2 | Hypothesized 4-factor 20 item model | 743.85 | 164 | 0.080 | 0.870 |
| AAP-P3 | 2-factor 16-item model informed by EFA | 504.20 | 103 | 0.084 | 0.889 |
| AAP-P3R | 2-factor 12-item informed by EFA | 225.36 | 53 | 0.077 | 0.932 |
| Analyses of cross-validation sample | | | | | |
| AAP-F3R | 2-factor 12- informed by EFA | 394.12 | 53 | 0.062 | 0.953 |

*Note.* Pilot sample (*N* = 549). Cross-validation sample (*N* = 1,674). *df* =degrees of freedom. RMSEA = root mean square error of approximation. CFI = comparative fit index.

**Table F-2**

*Standardized Loadings and Standard Errors (SE) for One-Factor Baseline Model of the Attitudes Toward Firearm-Related Policy Scale (Model AAP-P1)*

| Item | Label | Loading | *SE* |
| --- | --- | --- | --- |
| AAP17 | Creating a national registry of people who own firearms. | 0.79 | 0.021 |
| AAP19 | Requiring a waiting period of 3 to 5 days before someone can buy a firearm. | 0.78 | 0.026 |
| AAP16 | Requiring universal background checks for all firearm sales | 0.78 | 0.028 |
| AAP05 | No one should own AR-15-style semiautomatic rifles. | 0.77 | 0.023 |
| AAP14 | Requiring a permit for concealed carry. | 0.75 | 0.027 |
| AAP13 | Raising the minimum age to buy any kind of firearm from 18 to 21. | 0.74 | 0.028 |
| AAP20 | Laws that allow law enforcement officers to temporarily remove firearms from dangerous | 0.73 | 0.033 |
| AAP15 | Banning people from open carry. | 0.72 | 0.024 |
| AAP18 | Requiring a mental health evaluation before someone can buy a firearm. | 0.72 | 0.031 |
| AAP04 | Firearm control laws could prevent firearm violence, like mass shootings, homicides, and suicide | 0.70 | 0.028 |
| AAP02 | Firearm control laws go against my Second Amendment rights. | -0.69 | 0.038 |
| AAP07 | People should be required to get firearm training before they can purchase a firearm. | 0.67 | 0.034 |
| AAP03 | Firearm control laws hurt law-abiding citizens. | -0.66 | 0.040 |
| AAP12 | Preventing people who have committed violent crimes from owning or using firearms. | 0.49 | 0.044 |
| AAP09 | Firearm laws would not work because firearms are already in circulation. | -0.48 | 0.044 |
| AAP01 | Only law enforcement and military members should have firearms. | 0.48 | 0.037 |
| AAP06 | People who commit crimes with firearms should get more severe sentences. | 0.37 | 0.047 |
| AAP11 | There are enough firearm laws, but they are not being enforced. | -0.30 | 0.052 |
| AAP08 | Firearm safety should be offered in schools. | -0.25 | 0.046 |
| AAP10 | There are many ways to get around firearm laws. | 0.04 | 0.052 |

*Note.* Analyses based on pilot sample (*N* = 546). Items are listed in order of decreasing absolute value of loadings within each factor.

**Table F-3**

*Standardized Loadings and Standard Errors (SE) for a Hypothesized Four-factor Model of the Attitudes Toward Firearm-Related Policy Scale (Model AAP-P2)*

| Item | Label | Loading | *SE* |
| --- | --- | --- | --- |
| Competency | |  |  |
| AAP13 | Raising the minimum age to buy any kind of firearm from 18 to 21. | 0.76 | 0.027 |
| AAP18 | Requiring a mental health evaluation before someone can buy a firearm. | 0.75 | 0.032 |
| *AAP07* | People should be required to get firearm training before purchase. | 0.71 | 0.032 |
| AAP08 | Firearm safety should be offered in schools. | -0.19 | 0.047 |
| Monitoring/Oversight | |  |  |
| AAP16 | Requiring universal background checks for all firearm sales | 0.80 | 0.025 |
| AAP19 | Requiring a waiting period of 3 to 5 days before someone buys firearm. | 0.80 | 0.024 |
| AAP14 | Requiring a permit for concealed carry. | 0.78 | 0.026 |
| *AAP17* | Creating a national registry of people who own firearms. | 0.76 | 0.023 |
| AAP06 | People who commit crimes with firearms should get severe sentences. | 0.40 | 0.047 |
| General Attitudes | |  |  |
| AAP02 | Firearm control laws go against my Second Amendment rights. | 0.91 | 0.016 |
| AAP03 | Firearm control laws hurt law-abiding citizens. | 0.90 | 0.017 |
| AAP04 | Firearm control laws could prevent firearm violence, like mass shootings, ... | -0.69 | 0.034 |
| AAP09 | Firearm laws would not work because firearms are already in circulation. | 0.67 | 0.030 |
| AAP11 | There are enough firearm laws, but they are not being enforced. | 0.49 | 0.042 |
| AAP10 | There are many ways to get around firearm laws. | 0.09 | 0.049 |
| Restrictions | |  |  |
| AAP05 | No one should own AR-15-style semiautomatic rifles. | 0.76 | 0.025 |
| AAP15 | Banning people from open carry. | 0.73 | 0.024 |
| AAP20 | Laws that allow law enforcement temporarily remove firearms dangerous people | 0.73 | 0.033 |
| AAP12 | Preventing people committed violent crimes from owning or using firearms. | 0.50 | 0.044 |
| AAP01 | Only law enforcement and military members should have firearms. | 0.46 | 0.040 |

*Note.* Analyses based on pilot sample (*N* = 546). Items are listed in order of decreasing absolute value of loadings within each factor. Model resulted in an improper solution (e.g., two factors had estimated correlations greater than 1.00).

**Table F-4**

*Geomin-Rotated Factor Loadings For 2-Factor Exploratory Factor Analysis (Model AAP-E1)*

| Item | Label | Loading | *SE* |
| --- | --- | --- | --- |
| Items with primary loadings on Factor 1 | |  |  |
| AAP16 | Requiring universal background checks for all firearm sales | 0.80 | 0.02 |
| AAP19 | Requiring a waiting period of 3 to 5 days before someone buys firearm. | 0.80 | 0.02 |
| AAP14 | Requiring a permit for concealed carry. | 0.78 | 0.03 |
| *AAP17* | Creating a national registry of people who own firearms. | 0.76 | 0.02 |
| AAP06 | People who commit crimes with firearms should get severe sentences. | 0.40 | 0.05 |
| Items with primary loadings on Factor 1 | |  |  |
| AAP16 | Requiring universal background checks for all firearm sales | 0.02 | 0.84 |
| AAP14 | Requiring a permit for concealed carry. | 0.01 | 0.80 |
| AAP19 | Requiring a waiting period of 3 to 5 days before someone can buy a firearm. | -0.07 | 0.76 |
| AAP07 | People should be required to get firearm training before they can purchase a firearm. | 0.06 | 0.76 |
| AAP20 | Laws that allow law enforcement officers to temporarily remove firearms from dangerous | -0.04 | 0.74 |
| AAP13 | Raising the minimum age to buy any kind of firearm from 18 to 21. | -0.06 | 0.73 |
| AAP18 | Requiring a mental health evaluation before someone can buy a firearm. | -0.08 | 0.69 |
| AAP12 | Preventing people who have committed violent crimes from owning or using firearms. | 0.17 | 0.66 |
| AAP17 | Creating a national registry of people who own firearms. | -0.33 | 0.55 |
| AAP06 | People who commit crimes with firearms should get more severe sentences. | 0.14 | 0.51 |
| AAP05 | No one should own AR-15-style semiautomatic rifles. | -0.37 | 0.51 |
| Items without clear pattern of loadings on either factor | |  |  |
| AAP15 | Banning people from open carry. | -0.45 | 0.40 |
| AAP10 | There are many ways to get around firearm laws. | 0.26 | 0.26 |
| AAP01 | Only law enforcement and military members should have firearms. | -0.31 | 0.24 |
| AAP08 | Firearm safety should be offered in schools. | 0.34 | 0.01 |

*Note.* Analyses based on pilot sample (*N* = 546). Items are listed in order of decreasing absolute value of loadings within each factor.

**Table F-5**

*Standardized Loadings and Standard Errors (SE) for Two-Factor 16-Item Model Informed by Exploratory Analysis (AAP-P3)*

| Item | Label | Loading | *SE* |
| --- | --- | --- | --- |
| Negative Attitudes Toward General Firearm Policies | |  |  |
| AAP02 | Firearm control laws go against my Second Amendment rights. | 0.91 | 0.016 |
| AAP03 | Firearm control laws hurt law-abiding citizens. | 0.91 | 0.017 |
| AAP04 | Firearm control laws could prevent firearm violence, like mass shootings, homicides, and suicide | -0.69 | 0.033 |
| AAP09 | Firearm laws would not work because firearms are already in circulation. | 0.67 | 0.030 |
| AAP11 | There are enough firearm laws, but they are not being enforced. | 0.49 | 0.042 |
| Positive Attitudes Toward Specific Firearm Policies | |  |  |
| AAP16 | Requiring universal background checks for all firearm sales | 0.83 | 0.022 |
| AAP19 | Requiring a waiting period of 3 to 5 days before someone can buy a firearm. | 0.80 | 0.025 |
| AAP14 | Requiring a permit for concealed carry. | 0.79 | 0.025 |
| AAP13 | Raising the minimum age to buy any kind of firearm from 18 to 21. | 0.76 | 0.026 |
| AAP20 | Laws that allow law enforcement officers to temporarily remove firearms from dangerous | 0.76 | 0.031 |
| AAP17 | Creating a national registry of people who own firearms. | 0.75 | 0.023 |
| AAP18 | Requiring a mental health evaluation before someone can buy a firearm. | 0.75 | 0.030 |
| AAP05 | No one should own AR-15-style semiautomatic rifles. | 0.72 | 0.026 |
| AAP07 | People should be required to get firearm training before they can purchase a firearm. | 0.71 | 0.030 |
| AAP12 | Preventing people who have committed violent crimes from owning or using firearms. | 0.55 | 0.043 |
| AAP06 | People who commit crimes with firearms should get more severe sentences. | 0.41 | 0.046 |

*Note.* Analyses based on pilot sample (*N* = 546). Correlation between factors = -.63. Items are listed in order of decreasing absolute value of loadings within each factor. The following items were not included in the model: AAP15, AAP10, AAP01, and AAP08).

**Table F-6**

*Standardized Loadings and Standard Errors (SE) for Revised Two-Factor 12-Item Model Informed by Exploratory Analysis (AAP-P3R)*

|  |  | Pilot sample  (*N* = 549) | | Cross-validation sample  (*N* = 1,674) | |
| --- | --- | --- | --- | --- | --- |
| Item | Label | Loading | *SE* | Loading | *SE* |
| Negative Attitudes Toward General Firearm Policies | |  |  |  |  |
| AAP02 | Firearm control laws go against my Second Amendment rights. | 0.91 | 0.016 | 0.92 | 0.008 |
| AAP03 | Firearm control laws hurt law-abiding citizens. | 0.91 | 0.017 | 0.92 | 0.009 |
| AAP04 | Firearm control laws could prevent firearm violence, like mass shootings, homicides, and suicide | -0.69 | 0.033 | -0.62 | 0.023 |
| AAP09 | Firearm laws would not work because firearms are already in circulation. | 0.67 | 0.03 | 0.65 | 0.018 |
| Positive Attitudes Toward Specific Firearm Policies | |  |  |  |  |
| AAP05 | No one should own AR-15-style semiautomatic rifles. | 0.72 | 0.026 | 0.68 | 0.016 |
| AAP07 | People should be required to get firearm training before they can purchase a firearm. | 0.71 | 0.030 | 0.69 | 0.020 |
| AAP12 | Preventing people who have committed violent crimes from owning or using firearms. | 0.55 | 0.043 | 0.56 | 0.026 |
| AAP13 | Raising the minimum age to buy any kind of firearm from 18 to 21. | 0.76 | 0.026 | 0.75 | 0.016 |
| AAP14 | Requiring a permit for concealed carry. | 0.79 | 0.025 | 0.84 | 0.012 |
| AAP16 | Requiring universal background checks for all firearm sales | 0.83 | 0.022 | 0.86 | 0.012 |
| AAP19 | Requiring a waiting period of 3 to 5 days before someone can buy a firearm. | 0.80 | 0.025 | 0.87 | 0.010 |
| AAP20 | Laws that allow law enforcement officers to temporarily remove firearms from dangerous | 0.76 | 0.031 | 0.77 | 0.017 |

*Note.* Correlation between factors = -.63 in both samples. The following items from the 16-item model were not included: AAP06, AAP11, AAP17, and AAP18.

# **Appendix G**

## Sensitivity Analysis Comparing ANCOVA Findings Using Manifest Variables versus Latent Variables

This appendix reports the results of sensitivity analyses of ANCOVAs examining mean differences on the beliefs measures across groups that differ on sex, ethnicity, and firearms-related variables. Whereas the analyses reported in the manuscript were based on manifest variables (subscale scores calculated by taking the mean across items), the sensitivity analyses were based on latent variables based on the final CFA models. The analyses using latent variables generally replicated the findings of the analyses of the manifest variables. More specifically, they showed the same pattern of significant effects for all but four of the 152 parameter estimates included in each analysis. The three exceptions are reported in a bold, underlined font in the tables.

**Table G-1**

*Standardized Differences in Subscale Means (d-coefficients) on Attitude Measures by Sex and by Ethnicity for Analyses of Manifest Variables Versus Latent Variables*

|  | Sex differences^a^ | Ethnic differences^b^ | | |
| --- | --- | --- | --- | --- |
| Measure and subscale | Men/Women | Black/White | Black/ Latine | White/Latine |
| General Attitudes Toward Firearms |  |  |  |  |
| Positive Attitudes Toward Firearms | 0.30***/0.30*** | -0.38***/-0.37*** | -0.11/-0.10 | 0.27***/0.27** |
| Beliefs About Defensive Firearm Use |  |  |  |  |
| Respect Enforcement | **0.15*/0.09** | 0.73***/0.79*** | 0.32*/0.41* | -0.40***/-0.38*** |
| Defensive Escalation | 0.32***/0.36*** | -0.03/-0.05 | 0.07/0.08 | 0.10/0.12 |
| Compensatory Force | 0.03/-0.03 | 0.17/0.18 | 0.06/0.12 | -0.11/-0.06 |
| Prescriptive Norms for Threat Response |  |  |  |  |
| for Defense and Protection | 0.22***/0.17* | 0.10/0.00 | 0.18/0.15 | 0.08/0.15 |
| for Reputation and Respect | -0.09/-0.11 | 0.24*/0.28* | 0.03/0.03 | -0.21*/-0.25* |
| Attitudes Toward Firearm Policy and Prevention | |  |  |  |
| Negative Attitudes Toward General Firearm Policies | 0.15*/0.17* | -0.18/-0.20 | -0.24*/-0.30* | -0.06/-0.10 |
| Positive Attitudes Toward Specific Firearm Policies | -0.46***/-0.46*** | -0.04/-0.05 | 0.01/0.01 | 0.05/0.06 |

*Note. N* = 1,662. Coefficients are reported separately based on analyses of manifest/latent scoring of the attitude and belief variables. Coefficients represent mean in first group listed minus mean in second group listed in the column heading divided by the pooled SD. Bold underlined values indicate discrepancy in results of significance tests based on manifest versus latent variables.

^a^*N* = 867 women and 794 men. ^b^Pairwise comparisons for ethnicity were limited to three most frequent groups. *N* = 1,039 non-Hispanic White, 298 Latinx, 202 non-Hispanic Black. The analysis sample included 67 non-Hispanic Asian and 55 non-Hispanic multiracial participants, but comparisons with these groups are not reported due to the small number of participants within these groups.

**p* < .05. ***p* < .01. ****p* < .001.

**Table G-2**

*Standardized Differences in Subscale Means (d-coefficients) on Attitude Measures Across Groups Differing in Firearm-Related Access and Practices for Analyses of Manifest Variables Versus Latent Variables*

|  | Firearm Access^a^ | | |  |  |
| --- | --- | --- | --- | --- | --- |
| Measure and subscale | Personal Ownership/  No Access | Personal Ownership/  Household Ownership | Household Ownership /  No Access | Carry: Yes/No^b^ | Unsafe storage  Yes/No^c^ |
| General Attitudes Toward Firearms | |  |  |  |  |
| Positive Attitudes Toward Firearms | 0.95***/0.98*** | 0.50***/0.52*** | 0.45***/0.47*** | 0.82***/0.87*** | 0.53***/0.56*** |
| Beliefs About Defensive Firearm Use |  |  |  |  |  |
| Respect Enforcement | -0.03/-0.08 | -0.10/-0.14 | 0.07/0.06 | 0.17/0.03 | -0.08/-0.18 |
| Defensive Escalation | 0.51***/0.59*** | 0.29**/0.35*** | 0.22*/0.25* | 0.52***/0.61*** | 0.69***/0.82*** |
| Compensatory Force | 0.28***/0.34*** | 0.27**/0.37** | 0.01/-0.03 | 0.38***/0.41** | 0.36**/0.44** |
| Prescriptive Norms for Threat Response |  |  |  |  |  |
| Defense and Protection | 0.54***/0.56*** | 0.39***/0.43*** | 0.14/0.13 | 0.55***/0.55*** | 0.54***/0.55*** |
| Reputation and Respect | 0.00/0.01 | 0.09/0.11 | -0.10/-0.11 | 0.34**/0.36* | 0.20/0.16 |
| Attitudes Toward Firearm-Related Policy | |  |  |  |  |
| Negative Attitudes Toward General Firearm Policies | 0.64***/0.64*** | 0.30**/0.29** | 0.34**/0.35** | 0.78***/0.81*** | 0.60***/0.53*** |
| Positive Attitudes Toward Specific Firearm Policies | -0.65***/-0.64*** | -0.29**/-0.24* | -0.35***/-0.40*** | -0.83***/-0.86*** | -0.53***/-0.56*** |

*Note*. Coefficients reported separately based on separate analyses of manifest/latent variables controlling for sex and ethnicity. Coefficients represent mean in first group minus mean in second group listed in the column heading divided by the pooled SD. Bold underlined values indicate discrepancy in results of significance tests based on manifest versus latent variable means.

^a^*N* = 505 firearm owners, 146 with access to a firearm in their home, and 1,010 who reported not owning or having access. ^b^*N*s = 178 firearm owners who reported that they carry a firearm, and 260 firearm owners who reported they do not carry a firearm. ^c^*N*s = 149 firearm owners who reported using unsafe storage practices, and 317 firearm owners who did not report unsafe storage practices.

**p* < .05. ***p* < .01. ****p* < .001

**Table G-3**

*Standardized Differences Between Subscale Means (d-coefficients) on Attitude Measures Across Groups Differing in Firearm-Related Perpetration, Victimization, and Witnessing for Analyses of Manifest Variables Versus Latent Variables*

| Measure and subscale | Firearm-Related Perpetration:  Yes/No^a^ | | Firearm-Related Victimization  Yes/No^b^ | Firearm-Related Witnessing  Yes/No^c^ |
| --- | --- | --- | --- | --- |
|  | Model 1 | Model 2 |  |  |
| General Attitudes Toward Firearms |  |  |  |  |
| Positive Attitudes Toward Firearms | 0.62***/0.62*** | 0.35***/0.34** | 0.14/0.14 | 0.24**/0.23** |
| Beliefs About Defensive Firearm Use |  |  |  |  |
| Respect Enforcement | 0.29/0.17 | **0.30*/0.20** | **0.20**/0.14** | 0.07/-0.03 |
| Defensive Escalation | 0.36***/0.39** | 0.21/0.22 | 0.15/0.17 | 0.29***/0.31*** |
| Compensatory Force | 0.30/0.26 | 0.24/0.18 | 0.09/0.11 | 0.06/0.03 |
| Prescriptive Norms for Threat Response |  |  |  |  |
| Defense and Protection | 0.23/0.22 | 0.07/0.06 | 0.04/0.08 | -0.01/0.01 |
| Reputation and Respect | 0.14/0.17 | 0.15/0.17 | 0.09/0.10 | 0.13/0.14 |
| Attitudes Toward Firearm-Related Policy |  |  |  |  |
| Negative Attitudes Toward General Firearm Policies | 0.42**/0.43** | 0.22/0.24 | 0.14/0.16 | 0.12/0.13 |
| Positive Attitudes Toward Specific Firearm Policies | -0.66***/-0.69*** | -0.45**/-0.49** | -0.20*/-0.21* | -0.13/-0.13 |

*Note*. Coefficients reported separately based on separate analyses of manifest/latent variables controlling for sex and ethnicity. Coefficients represent mean in first group minus mean in second group listed in the column heading divided by the pooled SD. Bold underlined values indicate discrepancy in results of significance tests based on manifest versus latent variable means.

^a^*N* = 88 who reported using a firearm against someone and 1,568 who did not. Model 2 controlled for owning a firearm; Model 1 did not. ^b^*N* = 375 who reported being threatened, shot at, or injured by a firearm, and 1,286 who did not. ^c^*N* = 229 who reported seeing someone else get shot at, and 1,432 who did not.

**p* < .05. ***p* < .01. ****p* < .001.

**Table G-4**

*Standardized Differences Between Subscale Means (d-coefficients) on Attitude Measures Across Groups Differing in Firearm-Related Friends and Family Influence Variables for Analyses of Manifest Variables Versus Latent Variables*

| Measure and subscale | Friends carry firearm: Yes/No^a^ | | Firearms in home growing up: Yes/No^b^ | |
| --- | --- | --- | --- | --- |
|  | Model 1 | Model 2 | Model 1 | Model 3 |
| General Attitudes Toward Firearms Measure |  |  |  |  |
| Positive Attitudes Toward Firearms | 0.85***/0.90*** | 0.69***/0.73*** | 0.66***/0.70*** | 0.31***/0.31*** |
| Beliefs About Defensive Firearm Use |  |  |  |  |
| Respect Enforcement | 0.21***/0.21** | 0.23***/0.23** | 0.12/0.13 | 0.09/0.09 |
| Defensive Escalation | 0.42***/0.48*** | 0.34***/0.38*** | 0.33***/0.38*** | 0.26***/0.29*** |
| Compensatory Force | 0.33***/0.41*** | 0.29***/0.36*** | 0.16*/0.19* | 0.10/0.17 |
| Prescriptive Norms for Threat Response |  |  |  |  |
| Defense and Protection | 0.56***/0.57*** | 0.48***/0.49*** | 0.35***/0.35*** | 0.23**/0.27*** |
| Reputation and Respect | 0.32***/0.38*** | 0.34***/0.40*** | 0.13*/0.15* | 0.19*/0.22* |
| Attitudes Toward Firearm-Related Policy |  |  |  |  |
| Negative Attitudes Toward General Firearm Policies | 0.70***/0.71*** | 0.60***/0.61*** | 0.53***/0.55*** | 0.18/0.16 |
| Positive Attitudes Toward Specific Firearm Policies | -0.55***/-0.53*** | -0.43***/-0.42*** | -0.49***/-0.49*** | -0.36***/-0.35*** |

*Note*. Coefficients reported separately based on separate analyses of manifest/latent variables controlling for sex and ethnicity. Coefficients represent mean in first group minus mean in second group listed in the column heading divided by the pooled SD. Bold underlined values indicate discrepancy in results of significance tests based on manifest versus latent variable means.

^a^*N* = 1,090 who reported at least some friends carried firearms, and 558 who did not. Model 2 controlled for owning a firearm, Model 1 did not. ^b^*N* = 843 who reported having firearms in their home growing up, and 814 who reported they did not. Model 3 controlled for personal firearm ownership or household firearm ownership, Model 1 did not.

**p* < .05. ***p* < .01. ****p* < .001.

**Table G-5**

*Standardized Differences Between Subscale Means (d-coefficients) on Attitude Measures Across Groups Differing in Firearm-Related Friends and Family Injury Variables for Analyses of Manifest Variables Versus Latent Variables*

| Measure and subscale | Peer/family firearm injury:  Yes/No^a^ | Peer/family firearm suicide:  Yes/No^b^ |
| --- | --- | --- |
| General Attitudes Toward Firearms Measure |  |  |
| Positive Attitudes Toward Firearms | -0.04/-0.05 | 0.31***/0.31*** |
| Beliefs About Defensive Firearm Use |  |  |
| Respect Enforcement | **0.18*/0.15** | 0.09/0.09 |
| Defensive Escalation | 0.09/0.09 | 0.26***/0.29*** |
| Compensatory Force | 0.05/0.04 | 0.10/0.17 |
| Prescriptive Norms for Threat Response |  |  |
| Defense and Protection | 0.04/0.10 | 0.23**/0.27*** |
| Reputation and Respect | 0.09/0.11 | 0.19*/0.22* |
| Attitudes Toward Firearm-Related Policy |  |  |
| Negative Attitudes Toward General Firearm Policies | -0.03/-0.02 | 0.18/0.16 |
| Positive Attitudes Toward Specific Firearm Policies | 0.08/0.09 | -0.36***/-0.35*** |

*Note*. Coefficients reported separately based on separate analyses of manifest/latent variables controlling for sex and ethnicity. Coefficients represent mean in first group minus mean in second group listed in the column heading divided by the pooled SD. Bold underlined values indicate discrepancy in results of significance tests based on manifest versus latent variable means.

^a^*N* = 335 who reported having a friend or a family member injured or killed due to violence involving a firearm, and injured seeing someone else get shot at, and 1,326 who did not. ^b^*N* = 263 who reported having a friend or a family member attempt or commit suicide using a firearm, and 1,396 who did not.

**p* < .05. ***p* < .01. ****p* < .001.
